# New aspects of the esophageal histology of the domestic goat (*Capra hircus*) and European roe deer (*Capreolus capreolus*)

**DOI:** 10.1002/vms3.555

**Published:** 2021-06-19

**Authors:** Justyna Sokołowska, Kaja Urbańska, Joanna Matusiak, Jan Wiśniewski

**Affiliations:** ^1^ Department of Morphological Sciences, Institute of Veterinary Medicine Warsaw University of Life Sciences ‐ SGGW Warsaw Poland; ^2^ Department of Food Hygiene and Public Health Protection, Institute of Veterinary Medicine Warsaw University of Life Sciences ‐ SGGW Warsaw Poland

**Keywords:** domestic goat, esophagus, European roe deer, histology

## Abstract

The present study examines the esophageal wall of animals from two distinct families of the *Ruminantia*: domestic goats and European roe deer. Five fragments were collected from the entire length of the esophageal wall in five goats and four roe deer and subjected to microscopic and morphometric analyses. All layers of the esophageal wall except the tela submucosa were found to be thicker in the goats. In both species, the esophagus was lined by parakeratinized stratified squamous epithelium, and the tela submucosa was deprived of glands along its entire length. However, the structure of the lamina muscularis mucosae was better developed in goats: it was found to be discontinuous in the proximal part, and then became fused in the cervical part, that is around the most proximal quarter of its length. In contrast, in roe deer, the lamina muscularis mucosae began as sparse, thin muscle bundles at the pharyngeal‐esophageal junction, which thickened and clustered further down the esophagus, but did not fuse. Our findings regarding the microscopic structure of the ruminant esophagus are not fully consistent with the widely‐accepted view and suggest that the histological structure of the esophagus demonstrates interspecies variation within this large suborder. More precisely, species‐specific differences can be seen regarding the presence of esophageal glands and parakeratinized epithelium, and in the organization of the lamina muscularis mucosae.

## Graphical abstract

The comparative analysis of histological structure of esophagus in two species: domestic goat (*Capra hircus*) and European roe deer (*Capreolus capreolus*) was performed. Results show lack of esophageal glands and species‐specific variations in structure of the lamina mascularis mucosae in small rumianants esophagus.

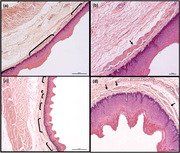

## 1 INTRODUCTION

The esophagus connects the pharynx with the stomach. While the general histology of the esophagus is well known (Zhang et al., 2018), the details of its microscopic structure vary depending on the species: variation has been found in the keratinization of epithelium, the presence and arrangement of the lamina muscularis mucosae, the presence and localization of the esophageal glands and in the histological structure of the tunica muscularis. Although these inter‐species variations in esophageal structure are widely known, some discrepancies exist between studies. For example, some histological textbooks state that the lamina muscularis mucosae is absent in dogs (Samuelson, 2007), whereas others indicate that it is present in the caudal region of the esophagus (Bacha & Bacha, 2006; Kuryszko & Zarzycki, 2000). Similarly, depending on the source, the submucosal esophagal glands in pigs have been reported to be localized in the first half of the esophagus (König & Liebich, 2007; Kuryszko & Zarzycki, 2000) or in its cranial part (Samuelson, 2007), or to gradually fade away along its length running anteriorly to caudally (Bacha & Bacha, 2006). Conflicting data also exist regarding the keratinization of the esophageal epithelium; for example, both keratinized (Bacha & Bacha, 2006) and non‐keratinized epithelia (Samuelson, 2007) have been described in pigs.

Studies comparing the histological structure of the esophagus between different animal species are not very numerous (Busch, 1980; Jamdar & Ema, 1982; Slocombe et al., 1982), and even fewer have been conducted on ruminants (Ebraheem et al., 2018; Islam et al., 2005; Islam et al., 2008; Kumar et al., 2009); therefore, most of the data regarding the esophageal histology of this suborder come from textbooks (Bacha & Bacha, 2006; Banks, 1993; König & Liebich, 2007; Kuryszko & Zarzycki, 2000; Samuelson, 2007). All treat ruminants as a single, homogeneous group of animals characterized by the same scheme of esophageal structure.

On the contrary, *Ruminantia* is a very large suborder of mammals that includes six families living in varied environmental conditions in almost all continents. Although domestic ruminants belong to the *Bovidae* family, they exist within two subfamilies: *Bovinae* (cattle) and *Caprinae* (goats, sheep). It is widely known that some anatomical differences exist between large and small domestic ruminants (König & Liebich, 2007; Kumar et al., 2018) and among various families of domestic and wild ruminants (Mbassa, 1988; Vidyadaran et al., 1999). As such, it is reasonable to assume that such differences may also concern the histological structure of the esophagus. Therefore, the aim of the present study was to compare the structures of the esophageal wall of two species, *viz*. the domestic goat (*Capra hircus*) and European roe deer (*Capreolus capreolus*), representing the *Bovidae* and *Cervidae* respectively, based on microscopic and morphometric analysis.

## 2 MATERIALS AND METHODS

### 2.1 Animals

Nine adult animals were included in the study: five domestic goats (3–5 years, 39–42 kg) and four European roe deer (approximately 3–4 years, 22–24 kg).

The goats were kept in confinement (i.e., a barn housing system). They were fed maize silage and crushed oats, which were administered once a day, and good quality meadow hay was provided each morning. Water and salt‐lick were constantly available to the animals *ad libitum*.

The study complied with Directive 2010/63/EU and the Act of the Polish Parliament dated 15 January 2015 on the protection of animals used for scientific purposes (Journal of Laws 2015, item 266). None of the animals were killed for the purposes of the study. All esophagi were collected from animal carcasses: the goats had been euthanized by pentobarbital overdose due to health problems (progressive arthritis, low milk yield), and the material was collected according to the rule of mutual sharing of organs and tissues for research purposes. For the European roe deer, the esophagi were collected from freshly‐hunted animals shot during collective hunting in January 2018 in the Puszcza Piska Forrest. The hunt took place in accordance with Polish hunting low (Act of the Polish Parliament dated 13 October 1995, item 713, the Hunting law, Chapter 3, Art. 8 Hunt), during the hunting season 2017–2018.

### 2.2 Tissue collection and preparation

The tissue specimens were collected within 1 h of animal death. All esophagi were removed together with pharynx. Incision of the distal end of esophagus was made just above the rumen inlet.

Five fragments of esophagus approximately 3 cm in length were collected from each animal. The first (cranial) included the area directly adjacent to the pharynx; the next samples were taken from the most caudal (caudal) and middle part of esophagus (middle). The last two fragments (mid‐cranial and mid‐caudal) were collected from the middle area of the proximal and distal parts of the esophagus that remained after collection of the first three fragments. Following this, each esophageal fragment was cut into two equal parts: the first was used for longitudinal sectioning and the second for transverse sectioning.

All esophageal fragments were fixed in 10% neutral buffered formalin, processed by the common paraffin technique and cut into 3 μm specimens. In total, 24 slides were prepared for each esophageal fragment from each animal: 12 longitudinal sections and 12 transverse sections. In each case the tissue specimens collected for analysis were separated from each other by a distance of 100 μm that is after obtaining each section 100 μm of paraffin block was cut and discarded. All specimens were stained with hematoxylin and eosin, Ayoub‐Shklar and Weigert‐Van Gieson methods. The Weigert‐Van Gieson method visualizes collagen and elastic fibers and differentiates connective tissue and muscle tissue (Bagiński, 1965). The Ayoub‐Shklar method differentiates keratin from other tissue components (Ramulu et al., 2013).

### 2.3 Microscopic and morphometric analysis

All tissue specimens were subjected to histological examination by light microscopy. In addition, the following individual morphometric measurements were made: the thickness of the epithelium, lamina propria and lamina muscularis mucosae, as well as the tela submucosa and tunica muscularis (thickness of each muscle layer was measured separately). The tunica adventitia was excluded from the analysis, because it was removed during tissue collection. Only tissue areas without any pathological lesions were included in the morphometric analysis.

For each animal, nine randomly‐selected specimens were analyzed from each esophageal fragment: three from the longitudinal sections and six from the transverse sections. In each of these specimens, measurements were performed in five randomly‐selected, equidistant microscopic fields, localized along the entire length of each longitudinal section, or along the perimeter of each transverse section. Each microscopic field was displaced from the previous one by 300 μm for the longitudinal sections and 500 μm for the transverse sections.

All specimens were viewed at a magnification of 100x. Morphometric analysis was performed under a Nikon Eclipse 80i microscope equipped with a Nikon DS‐Ri1 camera (Nikon, Tokyo, Japan) using NIS‐Elements BR microscope imaging software (Nikon). The micrographs were taken in all microscopic fields designed according to the algorithm described above. They represented a part of microscopic field at magnification of 100x with dimensions of 942.5 × 1178.4 μm. These images were used for morphometric analysis. Measurements perpendicular to the surfaces of particular layers were taken using specialized distance tools ('Horizontal' and 'Parallel lines'). As the thickness of the esophageal wall differed considerably not only between, but also within individual micrographs, especially in the transverse sections, three measurements were taken for each layer per micrograph. These values were used to calculate the mean thickness of each layer. This value was included in further analysis. The three measurements were taken at the thinnest and thickest points of the layer, and at a point of medium thickness. Where no considerable variation in esophageal wall thickness was observed, the measurements were taken from the opposite sides of the micrographs, with third one in the middle.

Thus, the following parameters were calculated: the thickness of each layer and the total thickness of the esophageal wall for each analyzed esophageal fragment and for the whole organ.

The results are presented as median and range (min‐max). As only a small number of individuals were tested, no statistical analysis was performed of the obtained data. All presented data refer to all the analysed animals within a single species. The measurements taken from individual animals are presented in Supplementary files.

## 3 RESULTS

**FIGURE 1:**
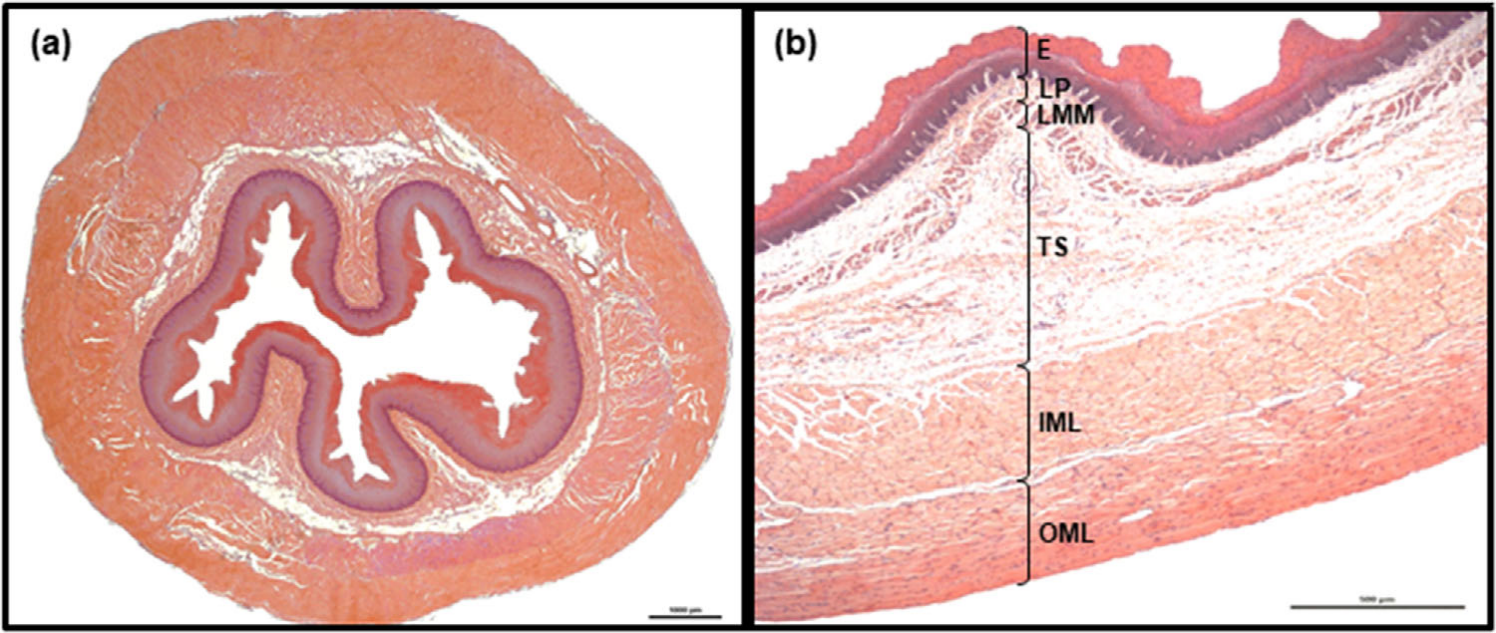
General histology of esophagus (a) and its wall with all layers marked (b) (transverse sections, goat). Tunica mucosa and tela submucosa create longitudinal folds giving the esophageal lumen a star‐like appearance (a). Hemotoxylin and eosin staining. Scale bars = 1000 μm (a) and 500 μm (b) Abbreviations: E, epithelium; IML, inner muscular layer; LMM, lamina muscularis mucosae; LP, lamina propria; OML, outer muscular layer; TS, tela submucosa.

The esophagi of both species were organized into distinct layers: tunica mucosa, tela submucosa and tunica muscularis (Figure 1). None of them contained obvious pathological lesions, except the presence of single Sarcocystis *spp*. scattered throughout the tunica muscularis in all roe deer.

All esophagi possessed longitudinal folds created by the tunica mucosa and tela submucosa. These folds were clearly visible on transverse sections, giving a star‐like appearance to the esophageal lumen (Figure 1a). Usually they were high, with deep grooves between them, pushing the epithelium into close proximity to the lamina muscularis mucosae.

**CHART 1:**
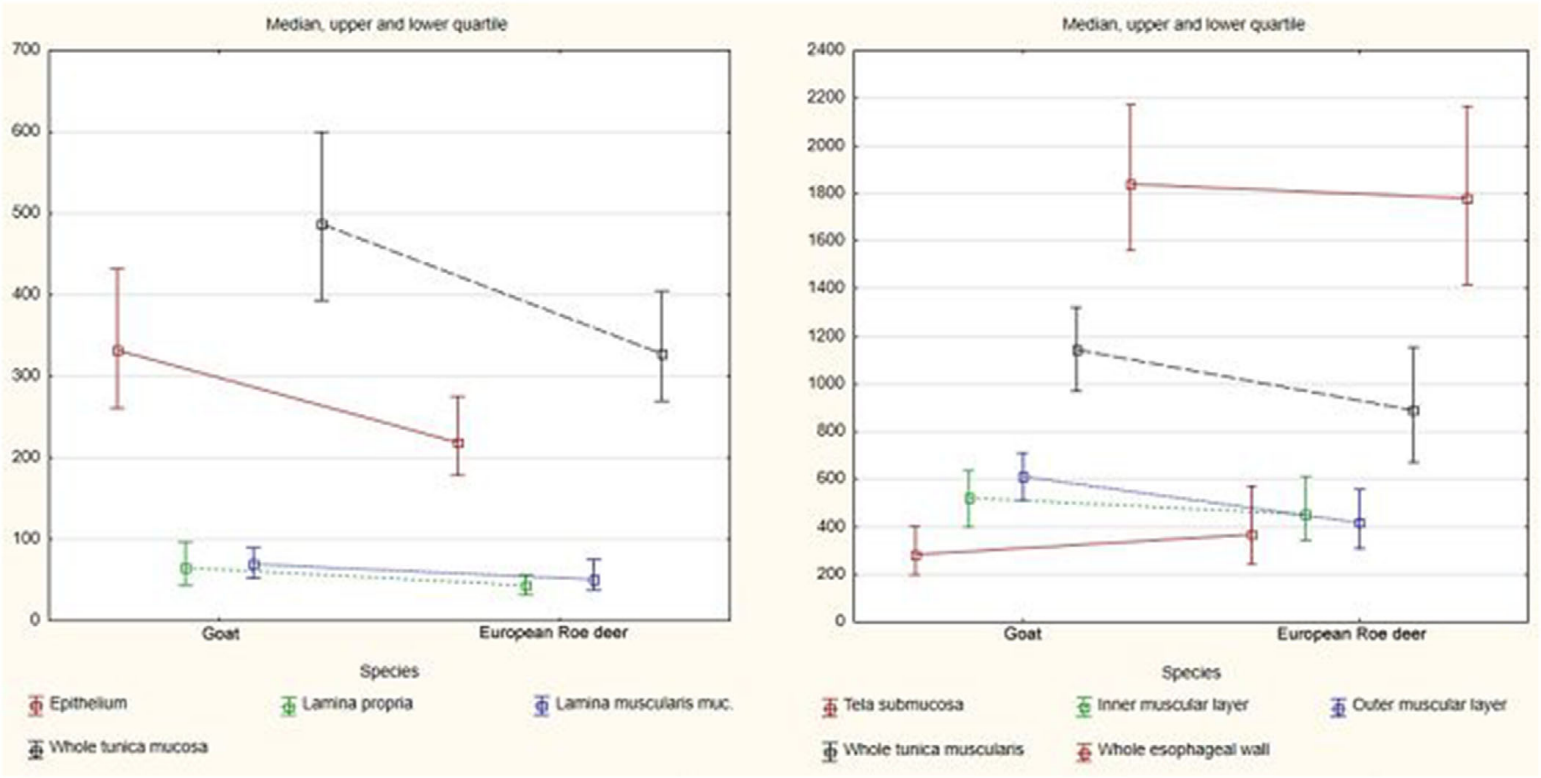
Comparison of median thickness of particular layers of esophagus between species

The median thickness of entire esophageal wall was 1840.9 μm (704.1–3999.8μm) in goats and 1778.4 μm (870.7–5111.6μm) in roe deer. All layers except the tela submucosa were thicker in goats. A graphical comparison of the thicknesses of particular layers of the whole esophagus with regard to species is given in Chart 1.

**FIGURE 2:**
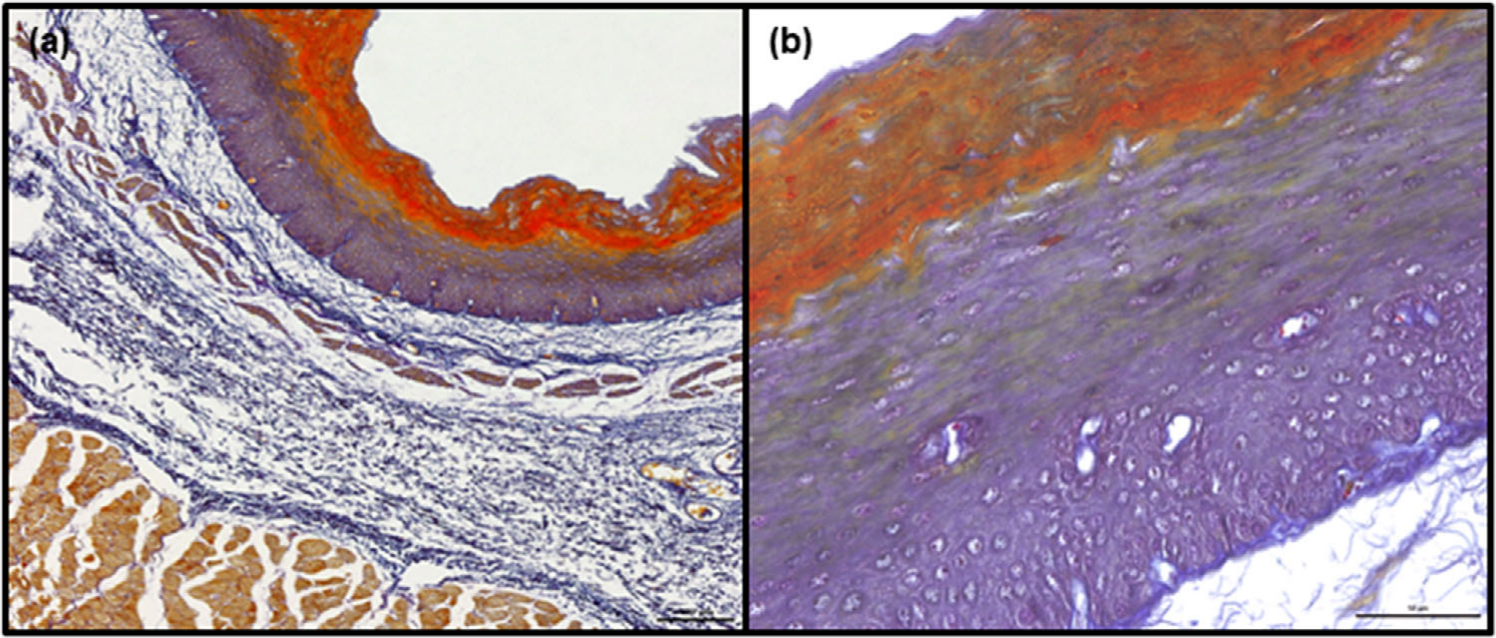
Representative images of parakeratinized esophageal epithelium (goat) in transverse section. The cells present in the superficial layers contain remnants of nuclei and keratin within their cytoplasm (brilliant orange). Ayoub‐Shklar staining. Scale bars = 100 μm (a) and 50 μm (b)

**Tunica mucosa**. In both species, the tunica mucosa consisted of typical layers: the epithelium, lamina propria and lamina muscularis mucosae. Also, in both species, the epithelium was the stratified squamous type. Remnants of nuclei were observed in the superficial layers of the epithelium; in addition, Ayoub‐Shklar staining confirmed the presence of keratin, indicating parakeratinization of the epithelium (Figure 2).

**FIGURE 3:**
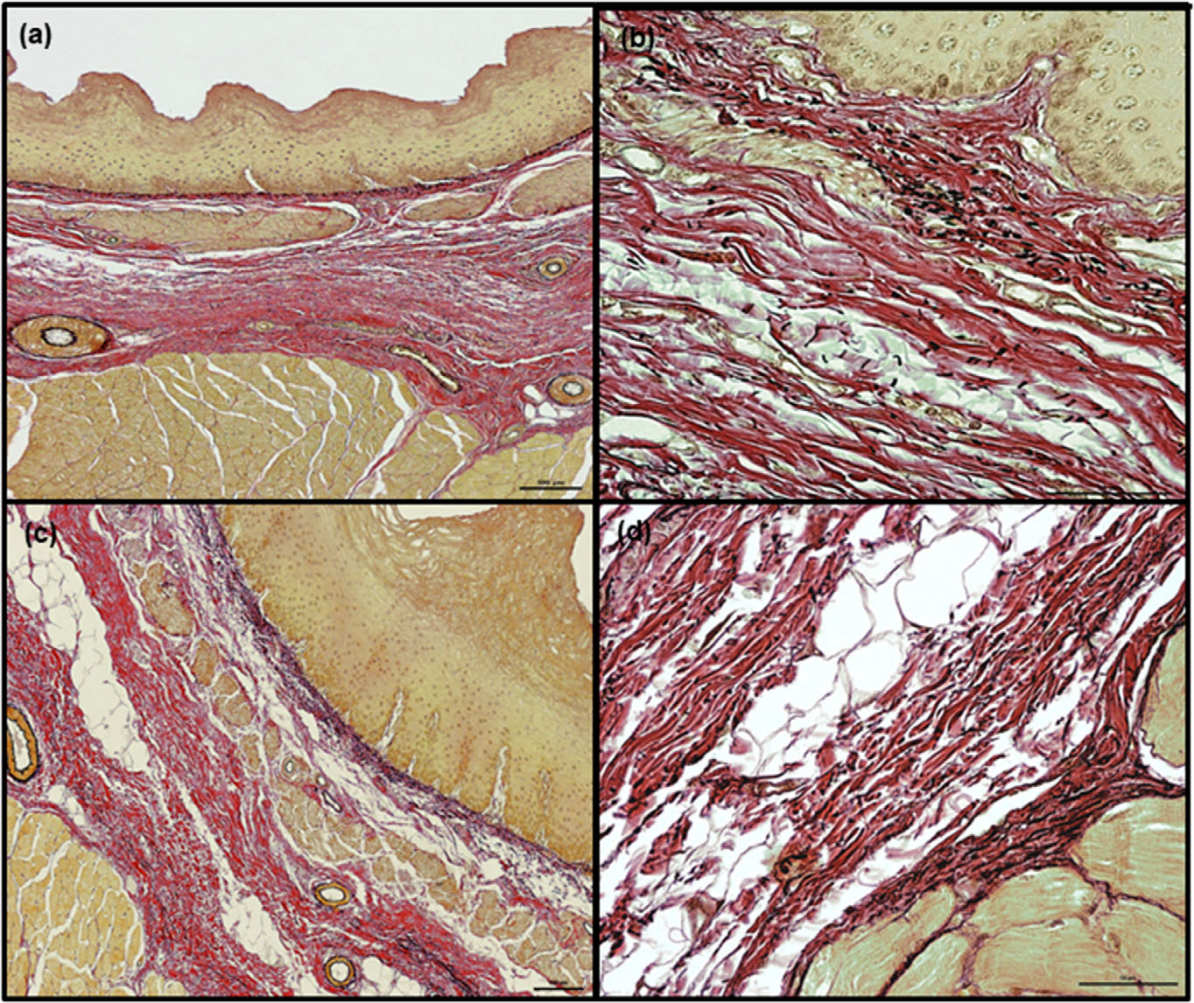
Localization of collagen and elastic fibers within lamina propria and tela submucosa of goat (a and b) and roe deer (c and d) esophagus in transverse sections. Elastic fibers (black) are the most numerous in lamina propria (a–c). The tela submucosa consists mainly of collagen fibers (red). In tela submucosa elastic fibers are sparse, slightly more numerous at the border of the tela submucosa and tunica muscularis (d). The muscles of lamina muscularis mucosae and tunica muscularis, as well as epithelium, are stained yellow. Weigert‐Van Gieson staining. Scale bars = 100 μm (a and c) and 50 μm (b and d)

**FIGURE 4:**
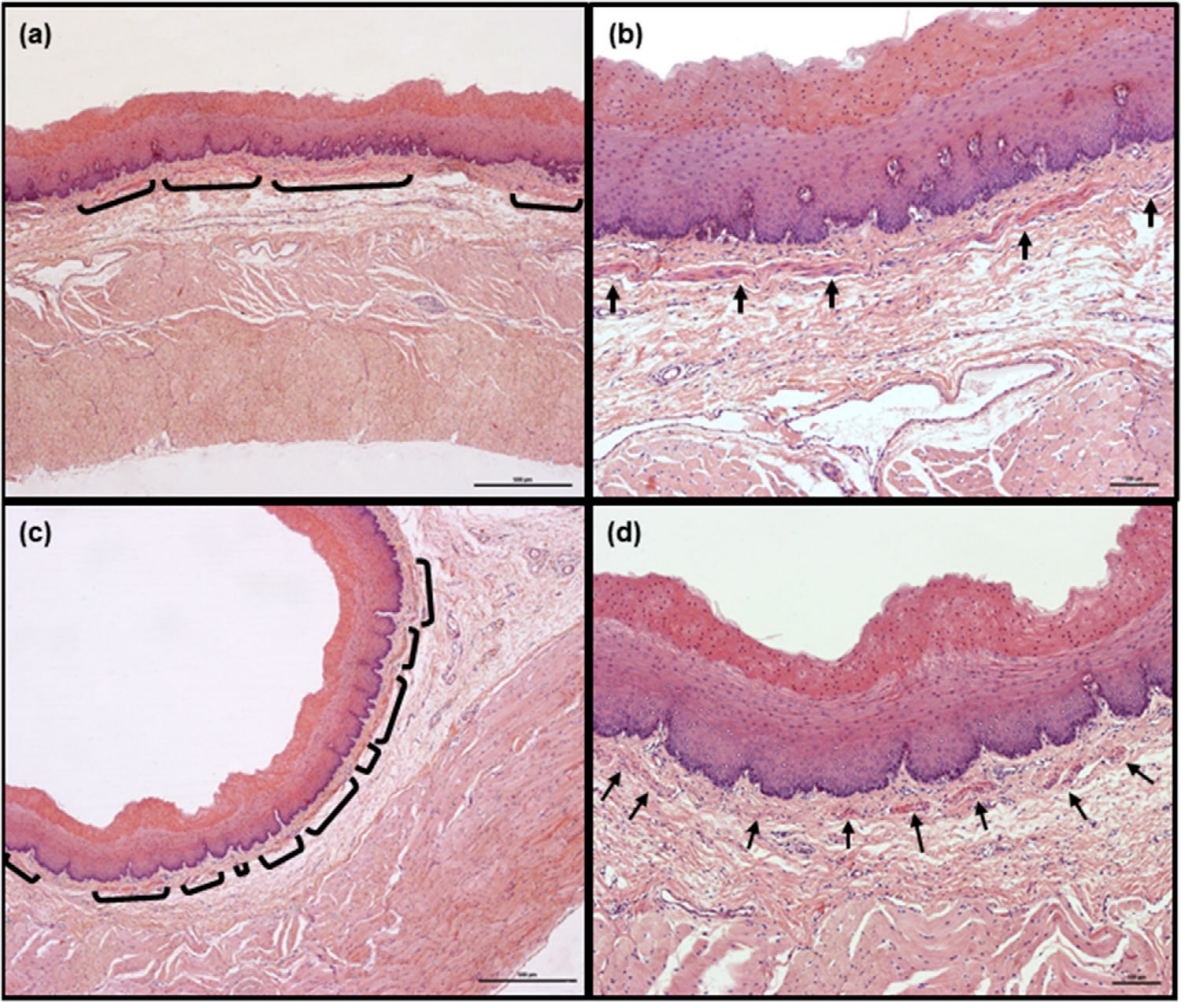
Representative image of cranial fragment of goat esophagus in longitudinal (a and b) and transverse (c and d) sections. Lamina muscularis mucosae is non‐continuous and consists of well‐developed smooth muscle bundles which, in transverse section, are small and are scattered irregularly, either separately or in small groups. Smooth muscle bundles are marked with brackets (a and c) or arrows (b and d). Hemotoxylin and eosin staining. Scale bars = 500 μm (a and c) and 100 μm (b and d)

**FIGURE 5:**
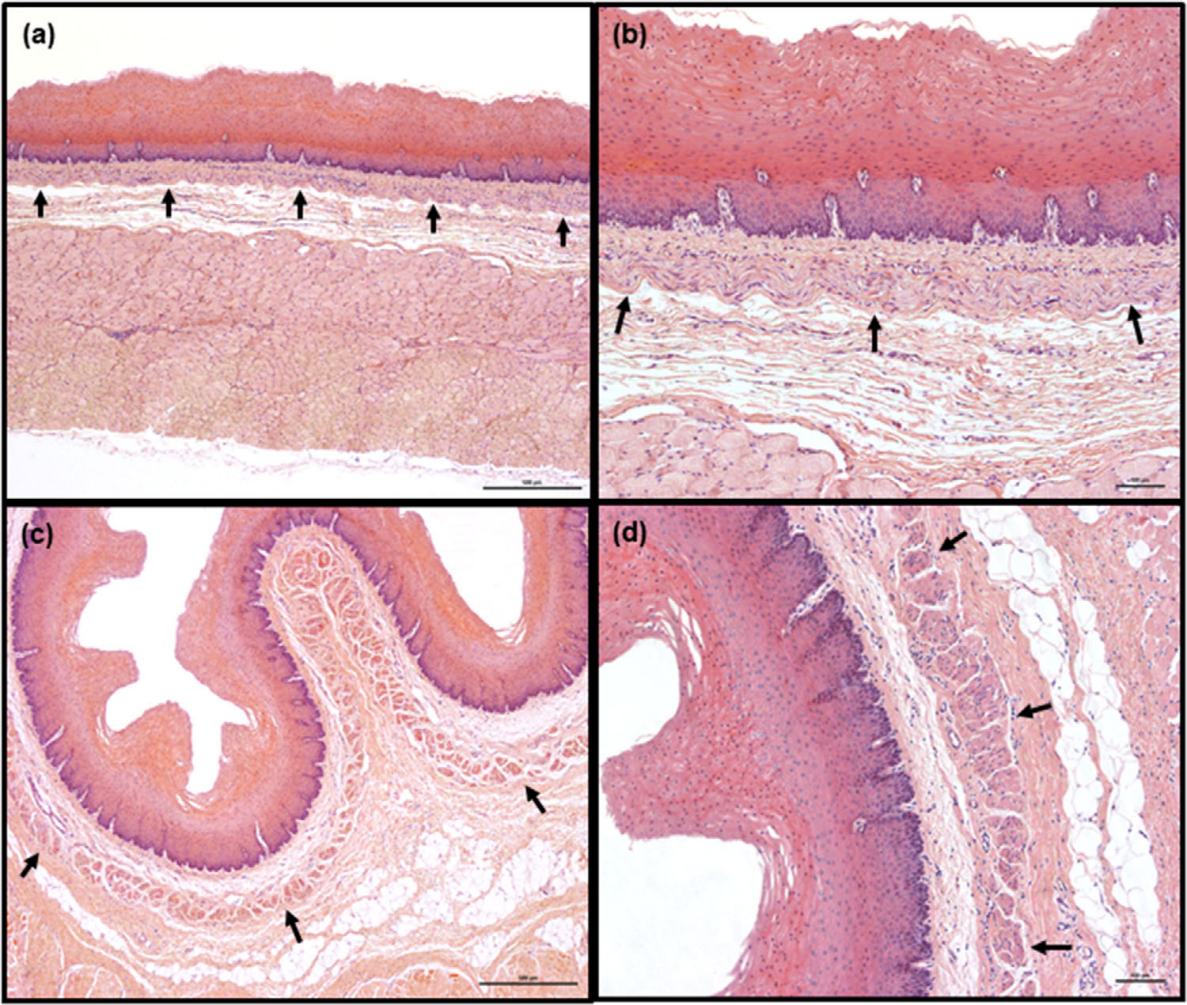
Representative image of caudal fragment of goat esophagus in longitudinal (a and b) and transverse (c and d) sections. The lamina muscularis mucosae (arrows) is continuous. Smooth muscle bundles (arrows) are thick and closely packed in transverse section. Large foci of adipocytes are visible in tunica submucosa (c and d). Hemotoxylin and eosin staining. Scale bars = 500 μm (a and c) and 100 μm (b and d)

**FIGURE 6:**
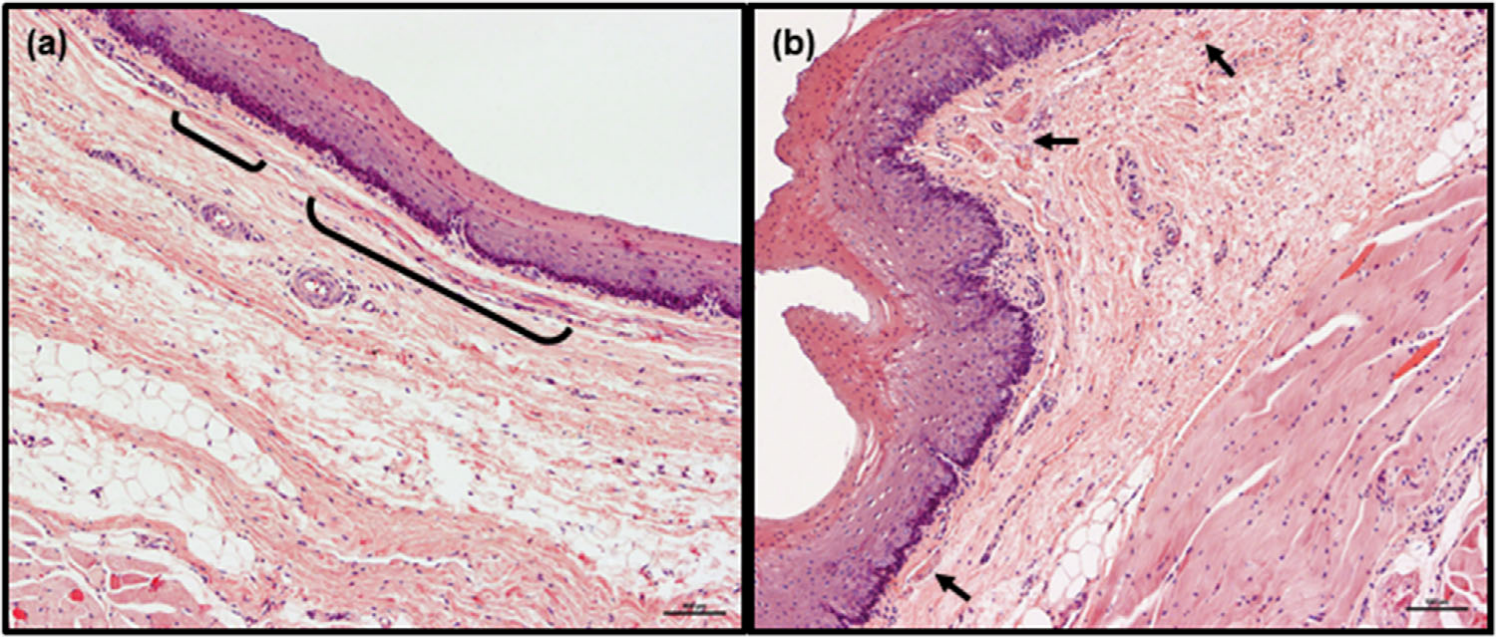
Representative image of cranial fragment of roe deer esophagus in longitudinal (a) and transverse (b) sections. Lamina muscularis mucosae is non‐continuous and consists of very thin muscle bundles which, in transverse section, are small and sparsely distributed. Smooth muscle bundles are marked with brackets (a) or arrows (b). Abundant adipose tissue is visible in tunica submucosa. Hemotoxylin and eosin staining. Scale bars = 100 μm

**FIGURE 7:**
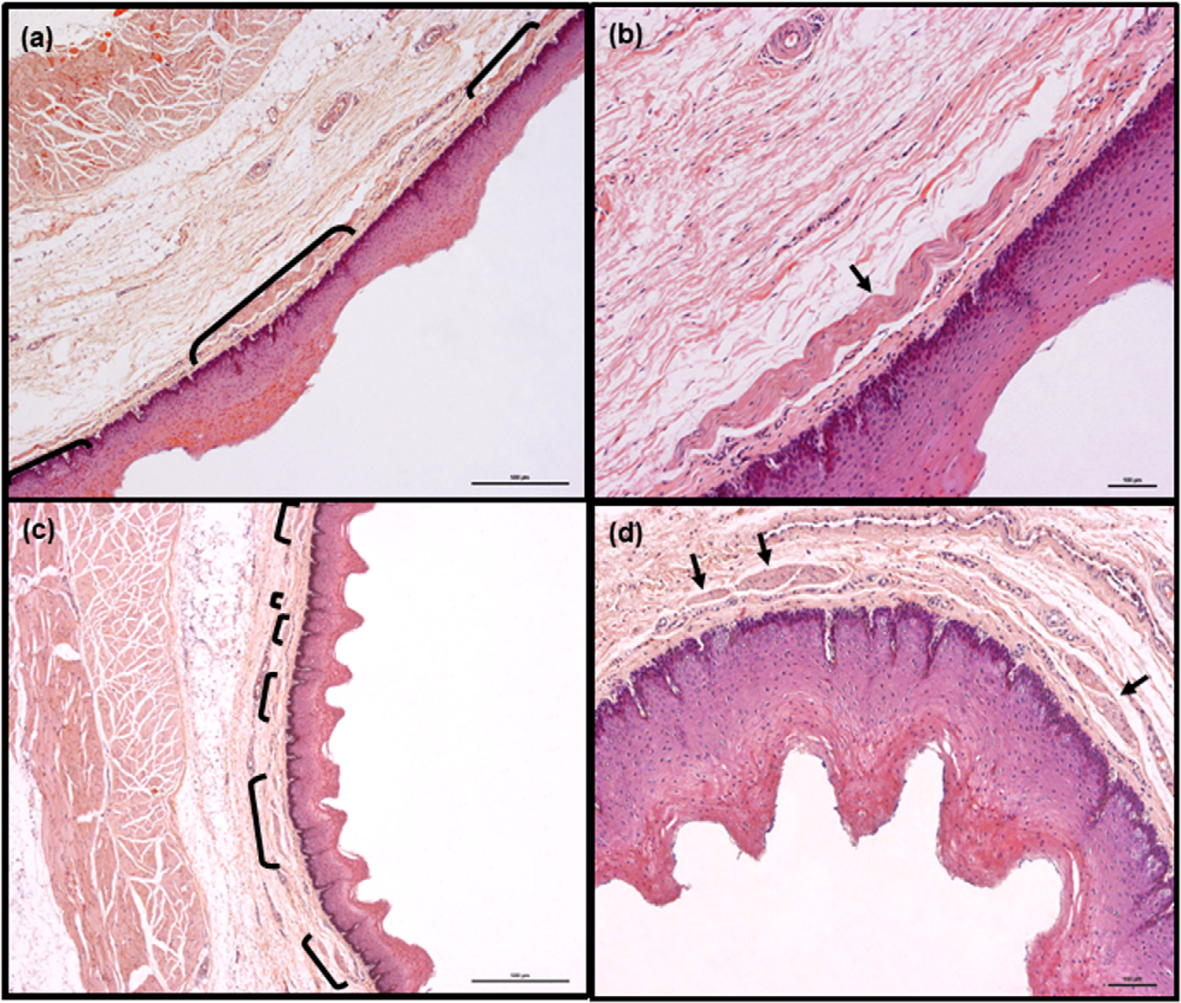
Representative image of mid‐cranial fragment of roe deer esophagus in longitudinal (a and b) and transverse (c and d) sections. Lamina muscularis mucosae is non‐continuous, but smooth muscle bundles are thicker and, in transverse section, located closer and usually arranged in groups. Smooth muscle bundles are marked with brackets (a and c) or arrows (b and d). Hemotoxylin and eosin staining. Scale bars = 500 μm (a and c) and 100 μm (b and d)

**CHART 2:**
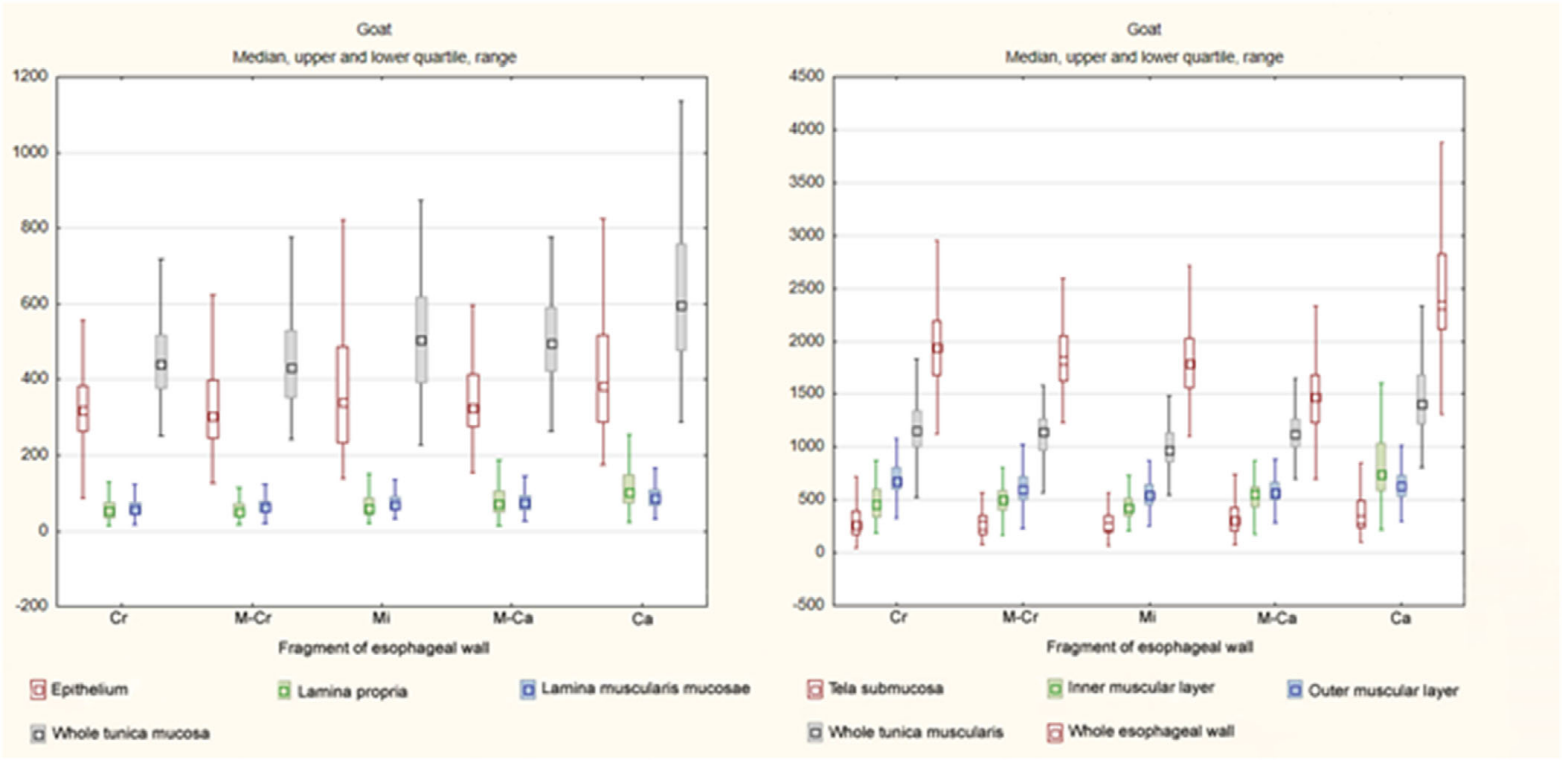
Comparison of median thickness of esophageal wall layers in particular fragments of esophagus in goats Abbreviations: Ca, caudal; Cr, cranial; M‐Ca, mid‐caudal; M‐Cr, mid‐cranial; Mi, middle.

**CHART 3:**
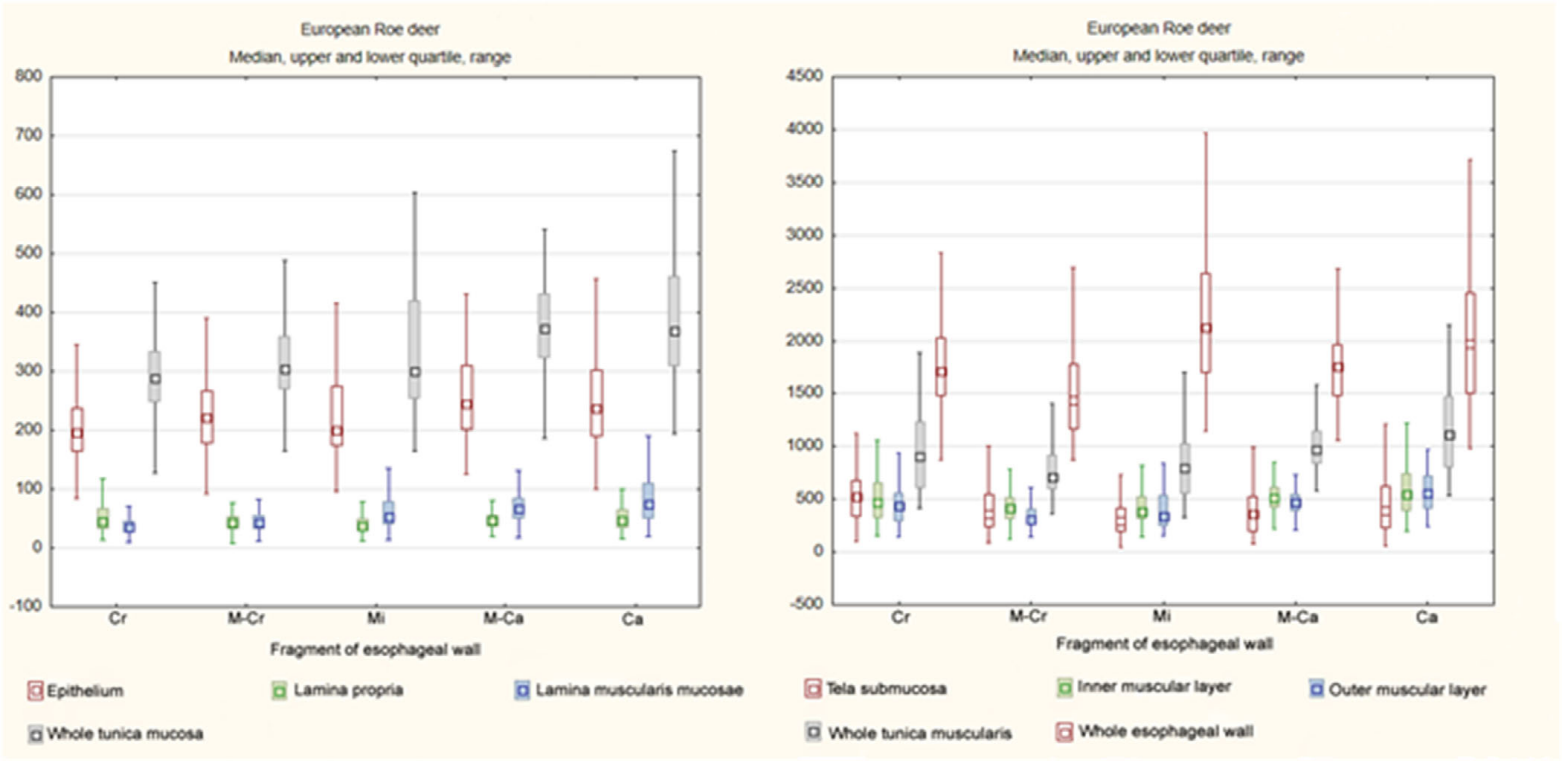
Comparison of median thickness of esophageal wall layers in particular fragments of esophagus in European roe deer Abbreviations: Ca, caudal; Cr, cranial; M‐Ca, mid‐caudal; M‐Cr, mid‐cranial; Mi, middle.

In both species, the epithelium was of unequal thickness (Figures 1b, 2a, 3a,c, 4d, 5c,d, 6b and 7c,d). The median thickness of the epithelium along the entire esophagus was 331.5 μm (61.7–1011.2 μm) in goats and 219.1 μm (84.3–1283.6 μm) in roe deer (Chart 1). In both species, the epithelium was of comparable thickness along the entire length of the esophagus; however, while the epithelium was thinnest in the mid‐cranial fragment and thickest in the caudal fragment in goats, it was thinnest in the cranial fragment and thickest in mid‐caudal fragment in roe deer (Table 1, Charts 2,3).

**TABLE 1:** Comparison of median thickness of esophageal wall layers in particular fragments of esophagus in goat and European roe deer

		Fragment of esophageal wall				
Thickness (μm)		Cranial	Mid‐cranial	Middle	Mid‐caudal	Caudal
**DOMESTIC GOAT**	Epithelium	319.2 (61.7‐779.3)	304.5 (126.4‐770.1)	339.1 (138‐823.3)	326.4 (155.1‐861.3)	382.9 (173.9‐1011.2)
	Lamina propria	52.7 (14‐219.4)	51.6 (18‐159.9)	59.9 (19.5‐222.5)	72.4 (13.7‐199.8)	102.4 (24.3‐359.2)
	Lamina muscularis mucosae	56.3 (15.9‐549.5)	62.2 (20.4‐151.7)	69.5 (32.7‐230)	75.3 (25.5‐181.6)	85.5 (32.2‐256.6)
	**Whole tunica mucosa**	**440.6** **(252.5‐1117.8)**	**430.2** **(243‐951.3)**	**505** **(226.2‐986.5)**	**496.5** **(262.7‐950.2)**	**596.3** **(289.4‐1225.3)**
	**Tela submucosa**	**267** **(48.6‐1243.8)**	**261.7** **(78.2‐1076.6)**	**257.4** **(62.4‐751.7)**	**308.3** **(82.2‐909.8)**	**319.1** **(97.6‐1116.3)**
	Inner muscular layer	454.5 (189‐1244.8)	502.5 (104.2‐804)	422.5 (207.2‐841.3)	557.7 (173.3‐974.7)	740.2 (224.8‐1600.6)
	Outer muscular layer	681.6 (161.8‐1640.4)	600.4 (225.6‐1219.7)	545.3 (247.9‐1022.3)	569.6 (287.9‐1073.1)	632.7 (295.5‐1200.1)
	**Whole tunica muscularis**	**1157.1** **(526.8‐2650)**	**1140.8** **(564.7‐1585.8)**	**971.8** **(546.1‐1661.7)**	**1122.7** **(576.2‐2008.5)**	**1405** **(803.6‐2329.5)**
	**Whole esophageal wall**	**1940.6** **(1125.9‐3713)**	**1820.2** **(1238.3‐2735.2)**	**1789.1** **(1101.8‐2712.1)**	**1471.5** **(704.1‐2333.6)**	**2344.4** **(1310.7‐3999.8)**
**EUROPEAN ROE DEER**	Epithelium	196.1 (84.3‐399.4)	221.3 (91.4‐537.4)	199.9 (97.1‐581.9)	245.9 (125.6‐432.3)	238 (99.7‐1283.6)
	Lamina propria	46.2 (12.9‐308.3)	42.5 (7.8‐114.7)	37.9 (11.8‐178.2)	46.3 (18.8‐155)	47.4 (16.3‐118.6)
	Lamina muscularis mucosae	35.2 (10.7‐97.8)	42.5 (12.5‐111.2)	53.7 (14.4‐193)	67.2 (17‐171)	74.7 (19.5‐277.6)
	**Whole tunica mucosa**	**288.6** **(126.9‐539.9)**	**304.9** **(165‐660.3)**	**299.3** **(165.3‐762)**	**371.7** **(186.7‐542)**	**368.7** **(193.8‐1581.3)**
	**Tela submucosa**	**520.2** **(101.8‐1201.5)**	**359.4** **(86‐1808.3)**	**295.6** **(50.9‐1276.2)**	**356.3** **(72.9‐1227.2)**	**394.4** **(59.2‐1305.7)**
	Inner muscular layer	469.8 (154.4‐1166)	412.7 (120.8‐787.8)	382.3 (138.5‐989.7)	515.5 (223.5‐1085.1)	551.7 (201.8‐1218.5)
	Outer muscular layer	439.2 (144.3‐1038.4)	311.6 (139.8‐961.5)	336.3 (158.2‐845.1)	466 (210.8‐1134.9)	558.7 (247.1‐975)
	**Tunica muscularis**	**907.3** **(411.3‐1882.2)**	**714.9** **(360.3‐1672.7)**	**795.4** **(327.8‐1697.3)**	**969.3** **(576.9‐2102.8)**	**1117.9** **(535.2‐2151.5)**
	**Whole esophageal wall**	**1717.4** **(870.7‐2972.8)**	**1435.5** **(874.8‐3063.2)**	**2130.8** **(1143.9‐5111.6)**	**1752.1** **(1058.4‐2976.6)**	**1978.3** **(985‐3714.7)**

All values are given as median (min‐max).

The lamina propria was formed by a thin layer of connective tissue with numerous elastic fibers (Figures 3a,b,c) and contained small blood vessels. No lymphatic nodules were observed. In goats, the median thickness of lamina propria along the entire esophagus was 65.4 μm (13.7–359.2 μm) (Chart 1); however, it was thinner in the first three fragments compared to the last two fragments, being thickest in the caudal fragment (Table 1, Chart 2). In the roe deer, the thickness remained comparable along the length of the esophagus; however, it was thinnest in the middle fragment, with a median value of 43.4 μm (7.8–308.3 μm) (Table 1, Charts 1,3).

**FIGURE 8:**
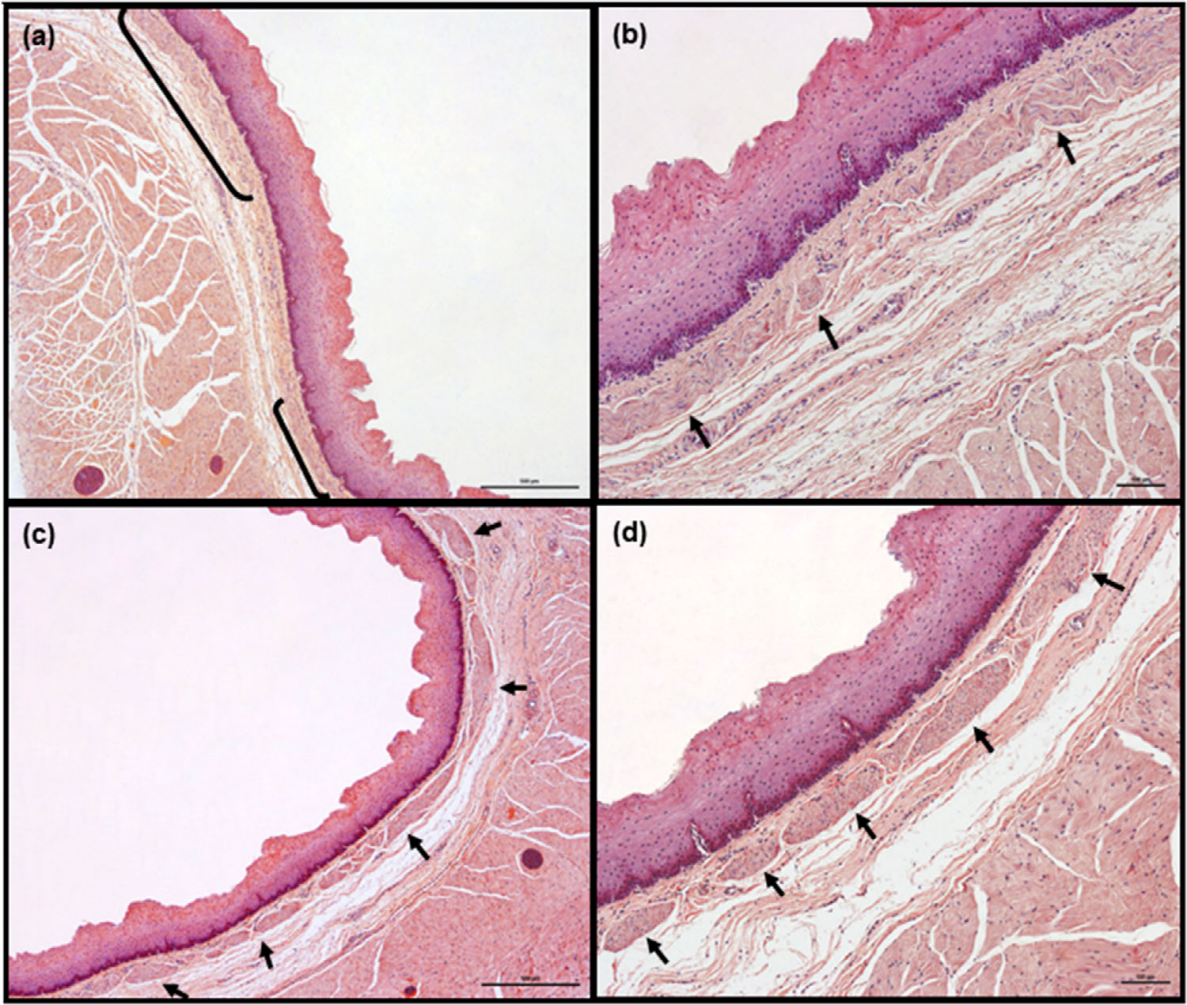
Representative image of caudal fragment of roe deer esophagus in longitudinal (a and b) and transverse (c and d) sections. The lamina muscularis mucosae is non‐continuous in longitudinal sections, but smooth muscle bundles appear thick and packed very closely in transverse section. Single Sarcocystis *spp*. are visible in tunica muscularis (a and c). Smooth muscle bundles are marked with brackets (a) or arrows (b, c and d). Hemotoxylin and eosin staining. Scale bars = 500 μm (a and c) and 100 μm (b and d)

**FIGURE 9:**
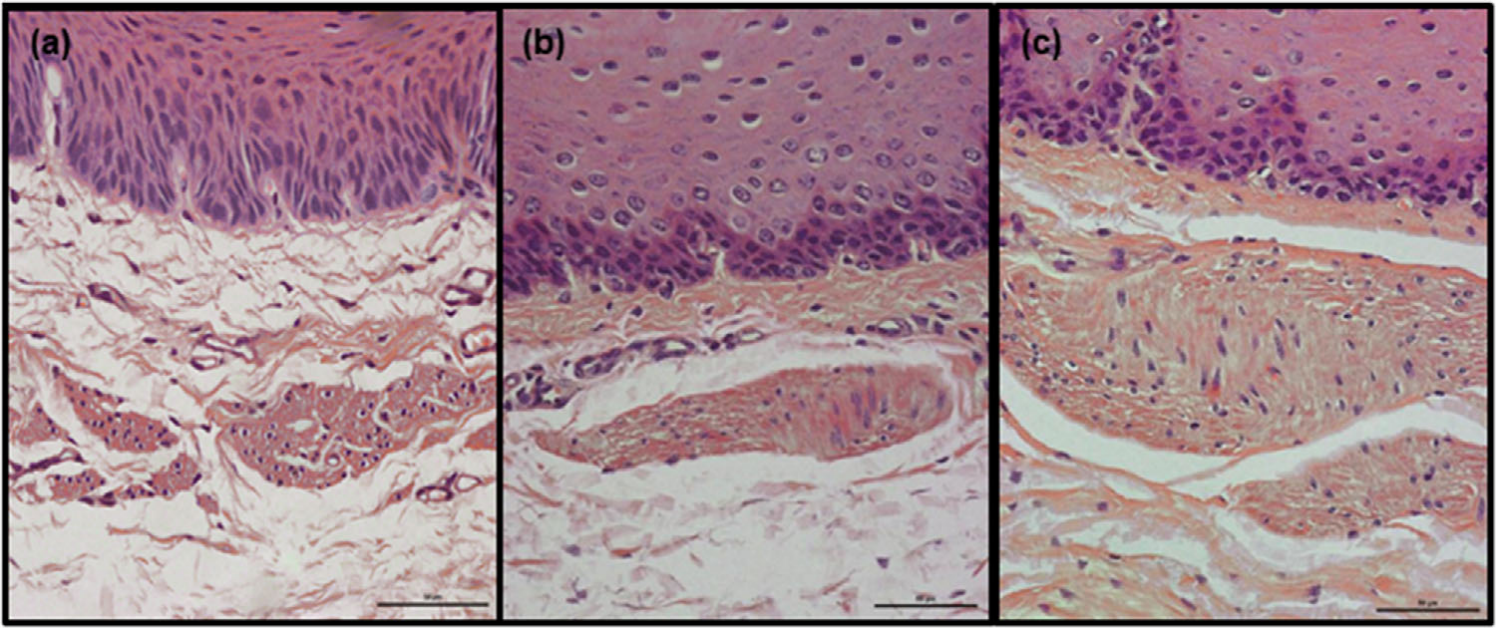
Representative images of lamina muscularis mucosae of goat (a) and roe deer (b and c) in transverse section. In both species, most muscle bundles consist of myocytes oriented longitudinally to the long axis of the esophagus (a). Certain bundles possess some muscle cells oriented vertically and localized in lateral (b) or central (c) part of bundle. Hemotoxylin and eosin staining. Scale bars = 50 μm

Obvious species‐specific differences were observed in the structure of lamina muscularis mucosae. It was composed of several layers of smooth muscle cells (from 2–3 to 5–6, depending on the species and exact fragment of the esophagus). On the longitudinally cut specimens, the lamina muscularis mucosae formed a compact, frequently wavy‐shaped, layer oriented longitudinally to the long axis of the esophagus (Figures 4a,b, 5a,b, 6a, 7a,b and 8a,b). In the transverse sections, the muscle cells were arranged in bundles of various shapes and diameters, separated by connective tissue and scattered around the entire perimeter of the esophagus (Figures 4c,d, 5c,d, 7c,d and 8c,d). Most of the muscle cells were oriented longitudinally to the long axis of esophagus; however, some cells demonstrated either a vertical or oblique orientation (Figure 9). Histologically, these muscle cells were not consistent elements in all muscle bundles. The smallest bundles were created by muscle cells oriented exclusively in one direction, that is, longitudinally to the long axis of the esophagus. Some larger bundles possessed various numbers of cells in vertical or oblique orientation localized either in the lateral or the central part of the bundle; these were scattered along the entire length of the esophageal wall among typical bundles.

In goats, the lamina muscularis mucosae was clearly recognizable in all parts of esophagus (Figures 4 and 5). Although it was non‐continuous in the cranial fragments, the distance between particular muscle bundles was relatively small in the longitudinal sections. In most animals, the bundles were long and well developed (Figures 4a,b); however, in one animal, they were shorter, less numerous and more widely spaced (data not shown). In transverse section, the lamina muscularis mucosae was visible as a relatively thin layer consisting of small muscle bundles lying individually or in small groups (Figures 4c,d). Some of them were separated by large areas of connective tissue, whereas others formed a relatively compact layer.

In the mid‐cranial fragments of the esophageal wall visible in longitudinal section, the lamina muscularis mucosae became continuous; however, in three animals, some muscle bundles were still separated by a short distance. In transverse section, the muscle bundles were larger and located closer than in the previous fragment of the esophageal wall. Moreover, this layer was more evident than in the cranial fragment (data not shown).

In the remaining fragments of esophagus, the lamina muscularis mucosae was arranged in a continuous manner in longitudinal section (Figures 5a,b). In transverse section, it formed a clearly‐visible continuous layer consisting of relatively closely‐packed muscle bundles (Figures 5c,d).

The median thickness of this layer throughout the whole esophagus was 69.7 μm (15.9–549.5 μm) (Chart 1). Its thickness progressively increased from the cranial to the caudal fragment (Table 1, Chart 2).

In roe deer, the lamina muscularis mucosae remained non‐continuous along its entire length. In the transverse section of the cranial fragment of the esophageal wall, the lamina muscularis mucosae of all animals could be seen to comprise small muscle bundles, which were sparsely and irregularly distributed within the connective tissue (Figure 6b). Although most were separate, some were arranged in small groups. Some differences in lamina muscularis mucosae structure were observed in longitudinal sections: in two animals, this consisted of only a few, relatively long but very thin muscle bundles localized sporadically in connective tissue (Figure 6a), while in another two, this arrangement was visible only in the proximal part of this fragment of esophageal wall, that is, in the first 3 or 5 of 12 consecutive tissue sections. In the remaining specimens, the muscle bundles were thicker and much closer together; such an arrangement was apparent in the mid‐cranial fragments of esophagi of all animals (Figures 7a,b).

In the transverse sections, the lamina muscularis mucosae of the mid‐cranial fragment was easier to recognize than that of the cranial fragment. The muscle bundles were larger than in the cranial fragment and usually grouped together (Figures 7c,d). Although they were still distinctly separated by connective tissue, they were closer together, with a consistent distance between them.

In the longitudinal sections of the middle fragments of the esophagi, the muscle bundles were arranged in a similar manner as in previous fragments; however, the bundles differed in length: in one animal, they were visibly longer than in other animals and were of comparable size to the muscle bundles observed in the mid‐caudal and caudal fragments, sometimes extending beyond the visual field at 100x magnification. In transverse section, the lamina muscularis mucosae was well developed and formed a continuous layer consisting of large, closely‐packed muscle bundles (data not shown).

In the longitudinal sections of the mid‐caudal and caudal fragments of the esophageal wall, the muscle bundles were longer than in previous fragments; however, they were of comparable length (Figures 8a,b). The gaps between them were either similar to those observed previously or smaller. In transverse section, the lamina muscularis mucosae in the mid‐caudal and caudal fragments had a similar appearance to the previous fragment (Figures 8c,d).

The median thickness of this layer was 51.6 μm (10.7–277.6 μm) along the entire esophagus (Chart 1), and the value progressively increased from cranial to caudal fragment (Table 1, Chart 3).

The median total thickness of the tunica mucosa in the entire esophagus was 487.1 μm (226.2–1225.3 μm) in goats and 328.4 μm (127–1581.3 μm) in roe deer (Chart 1). In goats, the tunica mucosa was thinnest in the mid‐cranial fragment and thickest in the caudal fragment (Table 1, Chart 2). In roe deer, it was thinnest in the cranial fragment and thickest in the last two fragments (which were of comparable thickness) (Table 1, Chart 3).

**Tela submucosa**. In both species, the tela submucosa consisted of connective tissue composed mainly of collagen fibers. These were interspersed with a small number of elastic fibers, scattered throughout the whole layer (Figure 3). The tela submucosa itself contained larger blood vessels, nerves and very sparse ganglia cells of submucosal plexus. Foci of white adipose cells, located close to the tunica muscularis, were observed. The adipocyte foci were mostly small and arranged in single cell rows, apart from in the most caudal fragment of the esophagus, where high amounts of adipose tissue were observed (Figures 5c,d). However, in one roe deer, large numbers of adipocytes were observed in the cranial fragment (Figure 6). Adipose tissue was more abundant in goats than in roe deer. No esophageal glands were observed in either species (Figures 1, 2, 3, 4, 5, 6, 7, 8).

In both species, the tela submucosa demonstrated great variations in thickness resulting from the presence of high longitudinal folds (Figure 1). The median thickness of the tela submucosa along the entire esophagus was 282.9 μm (48.6–1243.8 μm) in goats and 370.2 μm (51–1808.3 μm) in roe deer (Chart 1). In goats, the tela submucosa was thinner in three first fragments than the last two and was thickest in the caudal fragment. In roe deer, it was thinnest in the middle fragment and thickest in the cranial fragment (Table 1, Charts 2,3).

**Tunica muscularis**. In both species, the tunica muscularis was composed exclusively of skeletal muscle fibers (Figures 1 and 3, 4, 5, 6, 7, 8). They were arranged in two layers separated by poorly‐developed strands of connective tissue, containing ganglia cells of myenteric plexus, blood vessels and nerves. The muscle fibers were arranged in circular patterns in the inner layer and longitudinally in the outer layer. However, some focal disturbances in arrangement of muscle layers were observed in both species. In these areas, the border between the inner and outer layers was faded, and either both layers consisted of muscle fibers oriented in one direction, or the longitudinally‐arranged fibers of the outer layer were found to obliquely enter the inner layer. This phenomenon was observed throughout the whole length of esophageal wall (data not shown).

The median thickness of tunica muscularis along the entire esophagus was 1144.4 μm (526.8–2650 μm) in goats and 889.1 μm (327.8–2151.5 μm) in roe deer. The median thicknesses of the inner and outer muscular layers were 519.9 μm (104.2–1600.6 μm) and 611.3 μm (161.8–1640.4 μm) in goats and 453.9 μm (120.8–1218.5 μm) and 420.2 μm (139.8–1134.9 μm) in roe deer (Chart 1).

In goats, the tunica muscularis and its inner layer were thickest in the caudal fragment, whereas the outer layer was thickest in the cranial fragment. In contrast, the tunica muscularis and both its layers were thinnest in the middle fragment (Table 1, Chart 2). In roe deer, the whole tunica muscularis and its two layers were thickest in the caudal fragment, while the muscular layer was thinnest in the middle fragment of the esophagus (inner muscular layer) or the mid‐cranial fragment (outer muscular layer and whole tunica muscularis) (Table 1, Chart 3).

## 4 DISCUSSION

This study compared the histological structure of esophagus between two ruminant species: the domestic goat and the European roe deer. While some previous studies have examined the morphology of the esophagus in goats (Ebraheem et al., 2018; Islam et al., 2005; Islam et al., 2008; Kumar et al., 2009), no such studies appear to have been performed in roe deer. Our present approach is more detailed than the four studies on goats given above: whereas Ebraheem et al. (2018) include only one small sample of the anterior portion, and Kumar et al. (2009) only examine fragments of cervical, thoracic and cardiac segments of esophagus. Our analysis included samples collected from five different fragments of esophagus. In addition, these studies do not specify the mode that sections were taken, although the figures in the articles suggest that the slides were taken in transverse section. In contrast, each fragment in the present study was examined in both longitudinal and transverse section. Of the other two studies, Islam et al. (2008) focuses on anatomical localization of esophagus, whereas Islam et al. (2005) provide very short and superficial description of esophageal histology.

The present study also includes the first detailed morphometric analysis of all esophageal wall layers. Of the studies above, Islam et al. (2008) only measure the thickness of the whole esophageal wall, while Islam et al. (2005) only note the thickness of the main esophageal layers.

Our present findings reveal considerable differences in thickness of esophageal wall layers along the entire esophagus, especially in the tela submucosa. The structure of this layer allows the relatively inelastic mucosa to be pushed into longitudinal folds which obliterate the esophageal lumen, except during deglutition (König & Liebich, 2007). The thickness was frequently found to differ even as much as tenfold in the case of high folds with deep grooves. The differences in thickness of tunica mucosa also reflects, to a large extent, its histological structure. The lamina propria protrudes at intervals into the epithelium to form dermal papillae, and these can protrude into as much as 50% of the epithelium in humans (Long & Orlando, 2002). This can explain the enormous range of thicknesses observed for esophageal layers in our present analysis.

However, in carcasses, the thickness of the esophageal wall can also be attributed to contraction of the esophageal musculature associated with rigor mortis. Generally, this phenomenon starts developing within 1–2 h after death (Shivpoojan, 2018); however, it has been observed at 12–15 h post‐mortem in beef carcasses (Bodwell et al., 1965). In wild‐tailed deer, rigor mortis usually develops in the jaw or of the neck 2 h after death, with a 75% chance of none or partial rigor mortis 1 h after death (Oates et al., 1984). As the present samples of roe deer esophagi were collected within 1 h after death, the impact of rigor mortis on the thickness of esophageal wall appears to be minimal. In addition, as the goats were euthanized by pentobarbital overdose, the skeletal muscles were unlikely to contract (Ingalls et al., 1996; Lapointe & Côté, 1999; Thesleff, 1956).

In contrast, the roe deer were shot during hunting, which would result in cadaveric spasm: a condition in which a group of muscles that were used profusely just before death becomes stiff and rigid immediately after death (Fierro, 2013; Madea, 2013). However, in these cases, this would have affected the skeletal muscles of musculoskeletal system, with only minimal impact to the muscles of the internal organs. In addition, cadaveric spasm very rarely involves the entire body (Fierro, 2013; Madea, 2013).

It is possible that the varying thickness of particular layers of esophageal wall in roe deer could have resulted from increased muscle tone created by stress. Hunting, as a stress factor, is known to increase cortisol concentration, and high serum cortisol levels can increase smooth muscle tone (Xiao et al., 2003) and influence the activity of skeletal muscles (Tosato et al., 2015). However, deer shot in the field has been found to have low or average serum cortisol concentrations (Smith & Dobson, 1990); furthermore, the epithelium and lamina muscularis mucosae were not strongly folded, and the degree of waviness was similar to that observed in goats, suggesting similar muscle tension.

Our study indicates that all the layers of the esophageal wall are thicker in goats; with the exception of the tela submucosa, which was thicker in roe deer. This can reflect facts that goats eat firmer food (thicker epithelium), and they are able to swallow food boluses of larger volume (thicker tunica muscularis) (Ebraheem et al., 2018). Furthermore, in both species, the tunica muscularis was relatively thick in the cranial fragment (especially the outer muscular layer in goats), but thinned in the next one or two fragments before expanding gradually in the final two fragments. Increased thickness of tunica muscularis in the cranial esophageal fragment can reflect the presence of muscles oesophagei longitudinales that bind the esophagus to adjacent tissues, and lie externally to the esophageal muscle layer (Schaller & Constantinescu, 2007). These muscles attach to the external surface of esophagus, thus they could increase the thickness of the outer muscular layer. Indeed, we found that the outer muscular layer was thicker in the cranial fragment than the mid‐cranial fragment, especially in goats. However, we did not observe any distinct additional muscle layer presented in this esophageal fragment or areas of outer layer with disturbed arrangements of muscle fibers indicating the presence of muscles oesophagei longitudinales. To clarify this issue, further study is needed.

However, our study only includes a small group of animals, and further studies on larger number of individuals are needed to confirm our morphometrical results.

Possibly our most surprising finding is that no esophageal glands were present in the tela submucosa of both examined species. All textbooks of veterinary histology indicate that ruminants possess esophageal glands, with only slight differences existing in their precise localization: either in the cervical region of the esophagus (Banks, 1993; Islam et al., 2005; Kuryszko & Zarzycki, 2000) or at the pharyngeal‐esophageal junction (Bacha & Bacha, 2006; König & Liebich, 2007). However, our findings indicate a complete absence of esophageal glands in any of the histological sections taken from either species, including in the cranial fragment, which was always collected from the area directly adjoining the pharynx. It is surprising that no esophageal glands were identified at the pharyngeal‐esophageal junction area, considering the large number of sections examined from the anterior esophagus. Although similar findings were noted in two previous studies on goats (Ebraheem et al., 2018; Kumar et al., 2009), these should be regarded as tentative, as the methodology only included one tissue specimen collected from the cervical part of esophagus and did not specify the exact area from which it was taken. Even so, Saxena and Klimbacher (2019) also report a lack of esophageal glands in samples from sheep and cattle. Taken together, these findings, and our present ones, raise the question of whether ruminant species have esophageal glands at all, and if so, whether all species have them.

A number of histology textbooks (Bacha & Bacha, 2006; Kuryszko & Zarzycki, 2000; Samuelson, 2007) indicate that the ruminant esophageal epithelium is cornified. This belief results from the widely‐accepted opinion that the degree of keratinization of the epithelium depends on the coarseness of the diet (Banks, 1993; König & Liebich, 2007), and being herbivores that ingest hard and dry food, it is reasonable to assume that ruminants possess a keratinized epithelium (Kuryszko & Zarzycki, 2000). However, our present observations did not indicate the presence of fully keratinised anucleated scale‐like cells (Deo & Deshmukh, 2018) anywhere in the esophagi originating from either species. Rather, the esophageal epithelium of both species appeared to be parakeratinized which is a form of epithelial keratinization (Rao et al., 2014). In humans, such parakeratinized stratified squamous epithelia, and more commonly orthokeratinized epithelia, are associated with masticatory mucosa such as the hard palate, gingiva, dorsal surface of the tongue and the masticatory surfaces of the dental arches in the edentulous mouth (Groeger & Meyle, 2019; Nanci, 2018). The presence of this form of keratinization on the skin is considered a disease state, such as psoriasis, where it typically indicates an increased rate of epidermal turnover (Nanci, 2018). Therefore, parakeratinization is believed to be a unique histological feature of a healthy oral cavity (Nanci, 2018): a highly‐keratinized masticatory mucosa serves to dissipate shearing forces to a greater degree than a more flexible non‐keratinized epithelium, which is also found on the esophagus (Squier & Kremer, 2001). Therefore, it is possible that in goat and roe deer esophagi, parakeratinization of the epithelium provide protection against rough food while maintaining greater flexibility than a highly cornified epithelium.

This is the first record of such parakeratinization in either the masticatory or esophageal mucosa of ruminants. However, such pattern of epithelial maturation has been observed in the rumen, either as a pathological condition (rumen acidosis) or linked to feed type (concentrated diet) (Bull et al., 1965; Steele et al., 2011). Our results suggest that parakeratinization could be a more common phenomenon in the ruminant digestive tract than previously thought; this is supported by previous descriptions of goat esophageal epithelium (Kumar et al., 2009) and images of goat esophagus (Ebraheem et al., 2018) that suggest the presence of epithelial parakeratosis.

Information regarding the exact structure of lamina muscularis mucosae in ruminants is sparse; however, the consensus is that it consists of longitudinally‐arranged smooth muscle cells, forming isolated, scattered muscle bundles towards the anterior part of the esophagus, and that these bundles fuse to form a continuous layer posteriorly (Bacha & Bacha, 2006; Banks, 1993; Kuryszko & Zarzycki, 2000). However, no detailed studies have been conducted on ruminant species.

Although our present findings partially fill this gap, not all of our observations are consistent with available data, and clear interspecies differences were found in the structure of the lamina muscularis mucosae. Our results indicate that the lamina muscularis mucosae is better developed and thicker in goats than in roe deer. In the roe deer, it starts from individual, thin muscle bundles which gradually become thicker, longer and move closer together, achieving its final form in the middle fragment of the esophagus, that is, the cervical region. In contrast, in the goats, the lamina muscularis mucosae consisted of thick, long, well‐developed muscle bundles throughout the entire length of the esophagus; however, while the bundles were separated by narrow gaps in the cranial fragment, this layer became continuous in more distal fragments. The area of transition was localized between cranial and mid‐cranial fragments, in cervical region of esophagus.

The changes in the structure of lamina muscularis mucosae refer not only to the size of the individual muscle bundles and distance between them but also to their number. The number of individual muscle bundles scattered along perimeter of esophagus gradually increased together with increasing of their size. Such arrangement of lamina muscularis mucosae may provide a wide enough room for the wide expansion of the esophageal lumen during swallowing of foods in its cranial fragment and maintaining tension against the pressure from the internal cavity in the remaining part of esophagus (Nagai et al., 2003).

Our findings confirmed the structure of goat lamina muscularis mucosae described by veterinary textbooks (Bacha & Bacha, 2006; Banks, 1993; Kuryszko & Zarzycki, 2000); however, our observations indicate that the area where the separated muscle bundles fuse to form a continuous layer appears to be significantly closer to the pharynx, that is, at around the most proximal 1/4 of the esophagus. In addition, our findings suggest that this arrangement of muscle bundles is not characteristic for all ruminant species, and that at least in European roe deer, this layer is non‐continuous throughout the length of esophagus.

Our examination of goat and roe deer esophagi confirms previous observations that the tunica muscularis in ruminants consists entirely of skeletal muscles arranged in two typically oriented layers (Bacha & Bacha, 2006; Banks, 1993; Islam et al., 2005; König & Liebich, 2007; Kumar et al., 2009; Kuryszko & Zarzycki, 2000; Samuelson, 2007).

The present study describes a comparative analysis of the histological structure of the esophagus in two species belonging to the *Ruminantia* suborder that is, domestic goats and European roe deer. Certain interspecies variations were observed, especially regarding the arrangement of lamina muscularis mucosae. Moreover, some discrepancies were identified between our findings and the widely‐accepted description of the ruminant esophagus presented in histology textbooks. The main differences refer to the presence of esophageal glands, keratinisation of the epithelium and the organization of the lamina muscularis mucosae. Our findings suggest that interspecies variations exist in this large suborder of mammals, and that its members do not share a single common scheme regarding the histological structure of the esophagus. Therefore, to gain a fuller understanding of the histology of the ruminant esophagus, further studies are needed based on more detailed microscopic examinations of the esophagi of other species of domestic and wild ruminants.

## CONFLICT OF INTEREST

The authors declare that there is no financial or other conflict of interest regarding the publication of this article.

## AUTHOR CONTRIBUTIONS

*Study conception and design*: Justyna Sokołowska and Kaja Urbańska. *Material collection*: Justyna Sokołowska and Jan Wiśniewski. *Data analysis and/or interpretation*: Justyna Sokołowska, Joanna Matusiak and Kaja Urbańska. *Drafting of the manuscript*: Justyna Sokołowska. *Critical revision of the manuscript*: Kaja Urbańska. *Approval of the final version of the manuscripts*: All authors.

### PEER REVIEW

The peer review history for this article is available at https://publons.com/publon/10.1002/vms3.555.

## Supporting information

SUPPORTING INFORMATION

## Data Availability

The data that support the findings of this study are available from the corresponding author upon reasonable request.
