# Supplementary material for: New aspects of the esophageal histology of the domestic goat (Capra hircus) and European roe deer (Capreolus capreolus)
Source: Vet Med Sci. 2021 Jun 19;7(5):1743–56. doi: 10.1002/vms3.555 (PMC8464298; doi:10.1002/vms3.555)

**Appendix**

Goats – numerical and graphical comparison of median thickness of esophageal wall layers in particular fragments of esophagus in individual animals

| **Thickness [μm]** | | **Fragment of esophageal wall** | | | | |
| --- | --- | --- | --- | --- | --- | --- |
|  |  | **Cranial** | **Mid-cranial** | **Middle** | **Mid-caudal** | **Caudal** |
| **GOAT 1** | Epithelium | 300.2  (61.7-697.2) | 414.5  (253.1-648.7) | 490.2  (311.5-633.1) | 394  (246.1-630.9) | 534.2  (292.3-1011.2) |
|  | Lamina propria | 56.6  (15.7-193) | 62.2  (24.3-109.5) | 56.3  (24-140.5) | 66.6  (33.9-118.4) | 77.7  (44.5-158.4) |
|  | Lamina muscularis mucosae | 82.8  (24-549.5) | 69.6  (36.6-151.7) | 85.8  (51.5-230) | 85.2  (46.3-122.3) | 76.8  (32.42-210.4) |
|  | **Whole tunica mucosa** | **494.8**  **(322.1-1117.8)** | **550.3**  **(366.9-866.6)** | **644.4**  **(449.6-774.8)** | **539.7**  **(380.6-777.1)** | **713.6**  **(377.9-1137.7)** |
|  | **Tela submucosa** | **371**  **(69.7-938.8)** | **263.6**  **(124.4-537.8)** | **174.3**  **(62.4-751.7)** | **303.5**  **(145-709.5)** | **381.4**  **(129.2-888.9)** |
|  | Inner muscular layer | 530.8  (289.7-1244.8) | 465.2  (301.3-632) | 491.6  (233.3-841.3) | 560.3  (225.6-794.5) | 672.9  (224.8-1290.2) |
|  | Outer muscular layer | 753.8  (332.9-1640.4) | 743.4  (401-1219.7) | 608  (309.4-820.4) | 515  (301.4-670.1) | 738.4  (433.2-1200.1) |
|  | **Whole tunica muscularis** | **1304.6**  **(694.3-2650)** | **1249.1**  **(859-1585.8)** | **1057.3**  **(565.7-1661.7)** | **1069.1**  **(736.4-1464.6)** | **1427.8**  **(803.6-2316.3)** |
|  | **Whole esophageal wall** | **2227**  **(1374.2-3713)** | **2081.2**  **(1532.9-2598.6)** | **1912.2**  **(1234.4-2712.1)** | **1413.5**  **(931.9-1779.1)** | **2567.7**  **(1310.7-3648)** |
| **GOAT 2** | Epithelium | 297.5  (212.4-401.2) | 380.6  (210.2-770.1) | 259.4  (138-497.5) | 423.9  (240.7-861.3) | 450  (257.8-962.3) |
|  | Lamina propria | 58.9  (24.1-171.8) | 41.7  (18-74.8) | 40.3  (19.5-83.5) | 45.7  (22.6-95) | 96.7  (40.3-213.7) |
|  | Lamina muscularis mucosae | 59.3  (28.5-152.8) | 52.1  (24.5-106.7) | 60.5  (32.7-140.9) | 65.7  (25.5-181.6) | 104.7  (61.1-254.7) |
|  | **Whole tunica mucosa** | **416.4**  **(284.6-561.7)** | **475**  **(299.3-876.4)** | **363.6**  **(226.2-577.1)** | **545.1**  **(340.4-950.2)** | 683.7  **(439.6-1185)** |
|  | **Tela submucosa** | **240.1**  **(125.9-595.6)** | **215.5**  **(105.6-616.1)** | **258.9**  **(70.4-543.7)** | **269.2**  **(124.4-815.3)** | **309**  **(199.5-850)** |
|  | Inner muscular layer | 438.6  (250.2-790.3) | 360.5  (104.2-605.2) | 382.3  (212.8-555.2) | 516.9  (173.3-974.7) | 607.7  (310.5-1179.9) |
|  | Outer muscular layer | 686.3  (481.1-1063.1) | 520.2  (292.6-974.3) | 453.8  (247.9-787.4) | 659.2  (357.8-1073.1) | 616.7  (464.6-893.9) |
|  | **Tunica muscularis** | **1140**  **(805.7-1827.2)** | **850**  **(564.7-1314.8)** | **844.2**  **(546.1-1322.5)** | **1134.5**  **(576.2-2008.5)** | **1246.5**  **(976.3-1779.4)** |
|  | **Whole esophageal wall** | **1870.9**  **(1356.9-2459.9)** | **1644.3**  **(1238.3-2104.4)** | **1477.5**  **(1101.8-2205.3)** | **1649.3**  **(704.1-2333.6)** | **2276.8**  **(1819.7-3350.7)** |

All values are given as median (min-max)

(continued)

| **Thickness [μm]** | | **Fragment of esophageal wall** | | | | |
| --- | --- | --- | --- | --- | --- | --- |
|  |  | **Cranial** | **Mid-cranial** | **Middle** | **Mid-caudal** | **Caudal** |
| **GOAT 3** | Epithelium | 370.7  (248.7-710.6) | 273.8  (184.2-482.5) | 254.4  (177.5-445) | 328  (229.5-582.2) | 444.1  (276.4-791) |
|  | Lamina propria | 61.1  (15.2-201.2) | 53.6  (22.1-120.7) | 52.9  (19.5-135.2) | 83.3  (27.3-136.7) | 215.5  (56.6-359.2) |
|  | Lamina muscularis mucosae | 59.9  (19.3-147.8) | 59.5  (31.4-89.6) | 82.8  (34.9-171.8) | 77.8  (33.2-138.4) | 97.1  (32.2-153.5) |
|  | **Whole tunica mucosa** | **513.5**  **(354.7-840.7)** | **387.4**  **(259.8-603.3)** | **422.3**  **(278.9-625.1)** | **489**  **(333.3-762.5)** | **747.6**  **(491.8-1225.3)** |
|  | **Tela submucosa** | **300.2**  **(96.7-1243.8)** | **215.5**  **(101.7-364.6)** | **354.9**  **(147.5-586.5)** | **260.5**  **(102.1-573.7)** | **671.3**  **(281.7-1116.3)** |
|  | Inner muscular layer | 482.5  (209.2-1158.5) | 554.1  (261.4-648.3) | 450.4  (207.2-666.3) | 557.7  (285.8-875.1) | 1039.1  (411.2-1467.4) |
|  | Outer muscular layer | 482.5  (425.6-1071.4) | 445.1  (225.6-758.6) | 577.4  (458.3-933.4) | 562.5  (287.9-811.8) | 640.9  (375.6-823.1) |
|  | **Whole tunica muscularis** | **1171.7**  **(813.3-1809.9)** | **926.3**  **(722-1401.6)** | **1020.8**  **(784.6-1458.8)** | **1124.2**  **(744.5-1436.4)** | **1575.3**  **(1084.2-1926.7)** |
|  | **Whole esophageal wall** | **2049.5**  **(1432.2-3063.3)** | **1560.5**  **(1294.9-2097.2)** | **1883.9**  **(1306.3-2286.5)** | **1391.2**  **(1011.9-1971)** | **2984.5**  **(2007.7-3999.8)** |
| **GOAT 4** | Epithelium | 342.9  (236.1-452.6) | 304.5  (196.9-648.3) | 591.1  (322.5-823.3) | 311  (215.3-521.7) | 246.8  (184.6-478.6) |
|  | Lamina propria | 43.3  (14-93.4) | 56.8  (26.4-159.9) | 53.9  (27.6-93.6) | 94.2  (31.4-190.3) | 75.1  (24.3-187.3) |
|  | Lamina muscularis mucosae | 39.6  (15.9-74.1) | 64.8  (20.4-136.5) | 58.8  (35-108.6) | 74.8  (40.3-152.4) | 78.9  (52.9-174.4) |
|  | **Whole tunica mucosa** | **433.2**  **(335-545.3)** | **429.8**  **(275.3-841.6)** | **698.1**  **(413.6-986.5)** | **473.2**  **(347-707.8)** | **412.9**  **(289.4-663.4)** |
|  | **Tela submucosa** | **264.7**  **(87.2-584.6)** | **321.8**  **(78.2-973.9)** | **235.9**  **(135.6-571.5)** | **331**  **(170.6-716.3)** | **236.5**  **(97.6-491.5)** |
|  | Inner muscular layer | 331.3  (244.9-691.9) | 567.3  (343.9-687.6) | 386.2  (214.9-649.8) | 574  (349.5-769.1) | 661.7  (351.8-778.4) |
|  | Outer muscular layer | 631.7  (161.8-1113.8) | 690.7  (418.5-924.6) | 574  (447.6-798.7) | 655.7  (524.4-874.2) | 558.5  (380.3-821.7) |
|  | **Tunica muscularis** | **954.4**  **(526.8-1509.6)** | **1238.2**  **(994.4-1508.6)** | **962.9**  **(786.1-1201)** | **1251.9**  **(1035.2-1487.7)** | **1261.2**  **(843.9-1588.1)** |
|  | **Whole esophageal wall** | **1675.2**  **(1390-2356.2)** | **1987.8**  **(1464.1-2573)** | **1930.8**  **(1458.2-2474.1)** | **1568.7**  **(1301.1-2204.1)** | **1947.7**  **(1371.1-2400.5)** |

All values are given as median (min-max)

(continued)

| **Thickness [μm]** | | **Fragment of esophageal wall** | | | | |
| --- | --- | --- | --- | --- | --- | --- |
|  |  | **Cranial** | **Mid-cranial** | **Middle** | **Mid-caudal** | **Caudal** |
| **GOAT 5** | Epithelium | 235  (161.9-779.3) | 206.8  (126.4-705.1) | 233.8  (159.6-356.8) | 271.7  (155.1-413) | 289.3  (173.9-413) |
|  | Lamina propria | 52.7  (15.6-219.4) | 57.6  (23.1-108.1) | 145.4  (74.1-222.5) | 109.9  (13.7-199.8) | 116.4  (73.2-335.3) |
|  | Lamina muscularis mucosae | 53.2  (18.8-158.2) | 65.9  (35-138.4) | 67.8  (48.9-135.9) | 81.1  (33.9-180.1) | 80.6  (37.5-256.6) |
|  | **Whole tunica mucosa** | **343.6**  **(252.5-1054.3)** | **331.6**  **(243-138.4)** | **472.8**  **(326.5-614.1)** | **461**  **(262.7-612.8)** | **477**  **(346.9-787.5)** |
|  | **Tela submucosa** | **238.1**  **(48.6-842.1)** | **291.5**  **(101.9-1076.6)** | **272.2**  **(166.2-546.7)** | **358.8**  **(82.2-909.8)** | **279.2**  **(123.6-994)** |
|  | Inner muscular layer | 523.4  (189-868.5) | 554.7  (247-804) | 427  (282.6-732.2) | 484.8  (273.4-675.8) | 1080.7  (462.5-1600.7) |
|  | Outer muscular layer | 661  (447.4-1000.6) | 621.6  (359.8-1021.4) | 471.8  (303.7-1022.3) | 491.2  (370.4-714) | 591.7  (295.5-872.6) |
|  | **Whole tunica muscularis** | **1230.4**  **(802.4-1696.8)** | **1192.9**  **(1014.6-1427.9)** | **1011.1**  **(675.3-1422.4)** | **1040.5**  **(788.5-1221.9)** | **1634.2**  **(1087.6-2329.5)** |
|  | **Whole esophageal wall** | **1893.5**  **(1125.9-2797.6)** | **1812**  **(1589.2-2735.2)** | **1825.1**  **(1361.1-2238.7)** | **1282.6**  **(881-2016.5)** | **2344.4**  **(2021.7-2329.5)** |

All values are given as median (min-max)

Goat 1


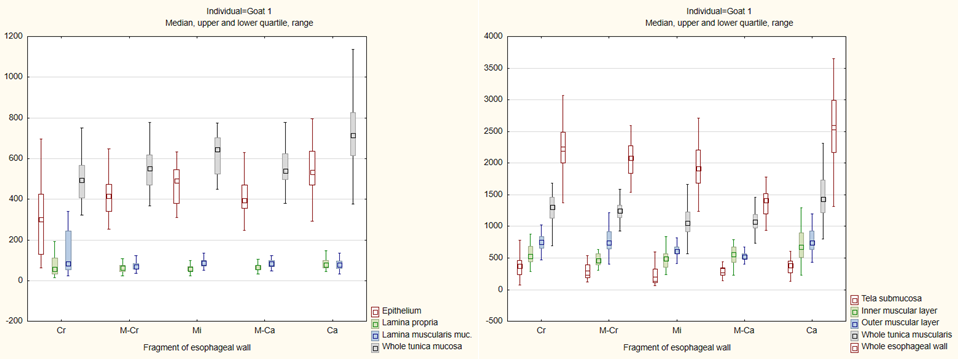


Goat 2


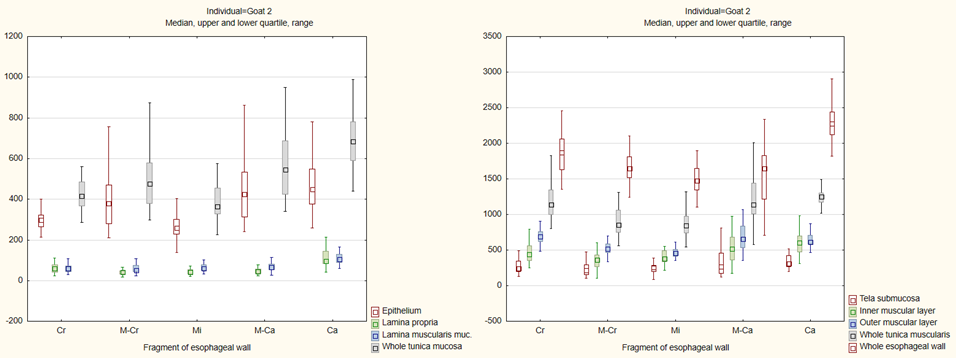


Goat 3


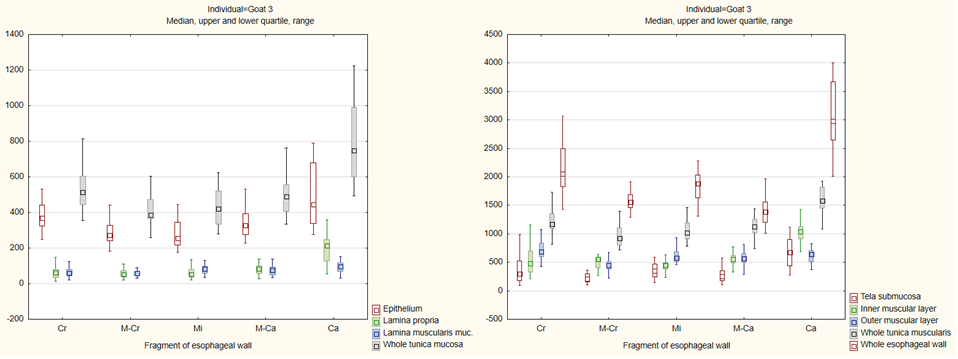


Goat 4


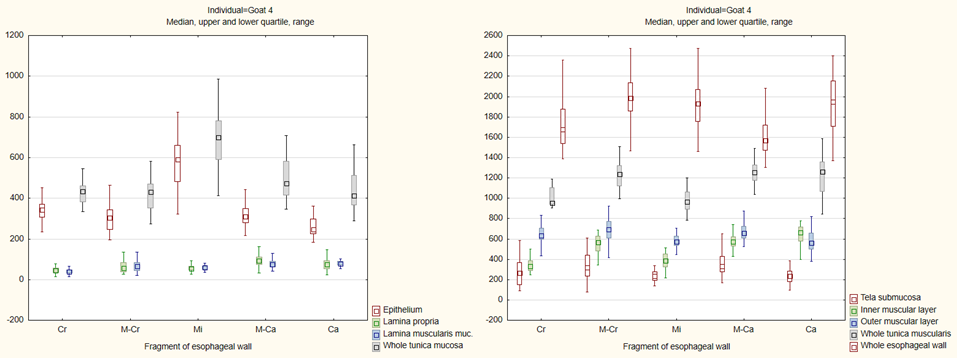


Goat 5


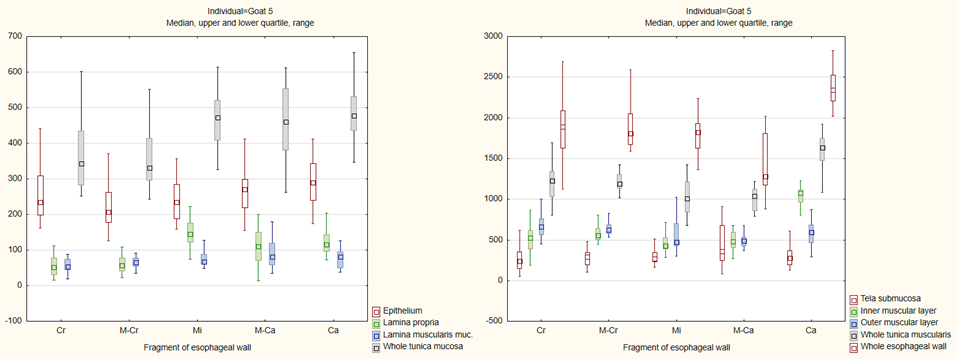


European roe deer – numerical and graphical comparison of median thickness of esophageal wall layers in particular fragments of esophagus in individual animals

| **Thickness [μm]** | | **Fragment of esophageal wall** | | | | |
| --- | --- | --- | --- | --- | --- | --- |
|  |  | **Cranial** | **Mid-cranial** | **Middle** | **Mid-caudal** | **Caudal** |
| **EUROPEAN ROE DEER 1** | Epithelium | 165  (84.3-382.7) | 175.8  (91.4-341.1) | 317.2  (172.6-581.9) | 257.1  (142.2-386) | 222.5  (99.7-1283.6) |
|  | Lamina propria | 29.4  (13.3-63.4) | 32.5  (18.2-84.6) | 42  (21.4-98.6) | 41.9  (23.1-80.5) | 48.3  (17.5-97.1) |
|  | Lamina muscularis mucosae | 44.2  (21.5-91.3) | 37.5  (12.5-111.2) | 77.9  (17.2-193) | 79.6  (17-163) | 107.2  (39.1-221) |
|  | **Whole tunica mucosa** | **251.7**  **(126.9-504.6)** | **249.2**  **(165-461.1)** | **481.7**  **(267.2-762)** | **393.8**  **(242.8-517.9)** | **396.5**  **(201.1-1581.3)** |
|  | **Tela submucosa** | **328.4**  **(101.8-1201.5)** | **209.8**  **(86-535.5)** | **242.7**  **(84.1-521.2)** | **303.5**  **(72.9-912.9)** | **369.5**  **(161.2-698.8)** |
|  | Inner muscular layer | 563.6  (308.8-931.8) | 424.5  (228.5-747.9) | 542.1  (138.5-989.7) | 566.7  (306.7-1085.1) | 660.3  (392-1175.6) |
|  | Outer muscular layer | 486.2  (227.7-766) | 275.4  (144.8-961.5) | 531.6  (300.2-733.9) | 468.1  (210.8-1017.8) | 631.6  (446.6-973.1) |
|  | **Whole tunica muscularis** | **1072.2**  **(653.7-1697.8)** | **649.2**  **(419.7-1672.7)** | **1023.8**  **(642.2-1570.6)** | **1059.2**  **(611.5-2102.8)** | **1304.5**  **(949.4-1998.8)** |
|  | **Whole esophageal wall** | **1652.3**  **(1042.8-2712.7)** | **1162.7**  **(874.8-2268.7)** | **2541.6**  **(1640.9-3296.2)** | **1846.2**  **(1058.4-2875.1)** | **2079.7**  **(1661.1-3714.7)** |
| **EUROPEAN ROE DEER 2** | Epithelium | 189.6  (102.8-399.4) | 245  (122-537.4) | 172.1  (115.5-247.9) | 259.9  (157.7-411.9) | 244  (139.2-456.2) |
|  | Lamina propria | 65.7  (27-135.1) | 41.5  (20.6-67.8) | 31.5  (19-59.9) | 46.3  (18.8-84) | 34.9  (16.3-69.9) |
|  | Lamina muscularis mucosae | 29.7  (10.7-61.3) | 35.3  (15.2-95.3) | 42.2  (14.4-86.4) | 57.4  (23.6-98.6) | 51.6  (19.5-140.3) |
|  | **Whole tunica mucosa** | **293.4**  **(174.7-486.3)** | **312**  **(201.9-660.3)** | **251.3**  **(175.6-336.8)** | **365.7**  **(252.8-503.7)** | **345**  **(193.8-558.8)** |
|  | **Tela submucosa** | **568.8**  **(141.4-1084)** | **292**  **(158.7-426.8)** | **131.4**  **(50.9-530.3)** | **189.7**  **(94.2-990.9)** | **153**  **(59.2-381.3)** |
|  | Inner muscular layer | 335.9  (154.4-716.1) | 291.9  (120.8-674.1) | 315.4  (194.1-403.3) | 490.7  (223.5-697.8) | 405.2  (240.7-756) |
|  | Outer muscular layer | 288.6  (144.3-579.3) | 264.1  (139.8-423.4) | 239.5  (171.6-392.1) | 429.1  (325.4-667.6) | 415.4  (247.1-708.9) |
|  | **Tunica muscularis** | **602**  **(420-1260.8)** | **556.9**  **(360.3-1097.5)** | **556.7**  **(389.4-795.4)** | **900.4**  **(730.8-1324.8)** | **829.6**  **(535.2-1429.2)** |
|  | **Whole esophageal wall** | **1519.6**  **(870.7-2580.7)** | **1172.2**  **(965-1987.2)** | **1327.6**  **(1143.9-2294.5)** | **1561.1**  **(1215-2383.3)** | **1324.7**  **(985-2055.8)** |

All values are given as median (min-max)

(continued)

| **Thickness [μm]** | | **Fragment of esophageal wall** | | | | |
| --- | --- | --- | --- | --- | --- | --- |
|  |  | **Cranial** | **Mid-cranial** | **Middle** | **Mid-caudal** | **Caudal** |
| **EUROPEAN ROE DEER 3** | Epithelium | 208.1  (163.1-377.7) | 215  (99.1-413.8) | 237.4  (148-372.7) | 229.6  (154.9-417.7) | 258.5  (166.6-438.5) |
|  | Lamina propria | 39.3  (22.1-81.5) | 44.1  (7.8-114.7) | 48.4  (24.3-178.2) | 46.3  (28.1-155) | 56.4  (25.2-118.6) |
|  | Lamina muscularis mucosae | 42.3  (24.5-97.8) | 54.7  (27.6-104.6) | 78.8  (40.8-164.8) | 78.9  (42.7-171) | 109.4  (44.3-277.6) |
|  | **Whole tunica mucosa** | **306.3**  **(212.5-485.8)** | **304.7**  **(198.9-560.1)** | **390.1**  **(241.3-603.3)** | **369**  **(258.6-540.1)** | **469.7**  **(308-690.3)** |
|  | **Tela submucosa** | **590.8**  **(253-1104.7)** | **484.6**  **(189.8-1808.3)** | **396.1**  **(189.3-1276.2)** | **357.1**  **(218.5-914.5)** | **697.2**  **(226.3-1045.9)** |
|  | Inner muscular layer | 689.5  (398.6-1166) | 541.3  (370.3-787.8) | 492.2  (314.2-940.4) | 547.6  (283.1-1047) | 786.6  (477.7-1218.5) |
|  | Outer muscular layer | 642.5  (373.1-1038.4) | 385  (243.5-740.9) | 540.3  (280.7-845.1) | 482.5  (272.4-1134.9) | 766.3  (507.4-975) |
|  | **Whole tunica muscularis** | **1346.1**  **(891.3-1882.2)** | **950**  **(637.7-1374.8)** | **1026.1**  **(757.5-1697.3)** | **1048.1**  **(576.9-2041.2)** | **1568.2**  **(1088.6-2151.5)** |
|  | **Whole esophageal wall** | **2195.8**  **(1556.8-2972.8)** | **1776.8**  **(1223.4-3063.2)** | **2746.9**  **(1830-5111.6)** | **1763.8**  **(1316.6-2771)** | **2691.8**  **(2070.5-3168)** |
| **EUROPEAN ROE DEER 4** | Epithelium | 197.7  (116.9-371.3) | 237.2  (175.4-389.7) | 187.8  (97.1-360.1) | 244.8  (125.6-432.3) | 221.6  (116.5-359.4) |
|  | Lamina propria | 56.9  (12.9-308.3) | 53.2  (33-86.3) | 35.8  (11.8-75.3) | 49.6  (26.8-90.2) | 45.8  (22.5-96.3) |
|  | Lamina muscularis mucosae | 29.6  (12.9-57.5) | 47.1  (29-98.7) | 41  (23.8-63.3) | 57.9  (21.4-93.6) | 55.4  (27.3-91.6) |
|  | **Whole tunica mucosa** | **302.5**  **(164.4-539.9)** | **337.8**  **(270-502.5)** | **264.3**  **(165.3-436.1)** | **365.2**  **(186.7-542)** | **350.7**  **(196.1-491.4)** |
|  | **Tela submucosa** | **631**  **(257.9-1121.9)** | **580.8**  **(327.2-1001.8)** | **341**  **(169.5-617.4)** | **485.6**  **(214.1-1227.2)** | **511.4**  **(253.2-1305.7)** |
|  | Inner muscular layer | 335  (167.3-723.7) | 401.3  (183-720.5) | 316.2  (152.3-459) | 451.8  (225.2-706.6) | 382.6  (201.8-732.9) |
|  | Outer muscular layer | 342.1  (195.3-583) | 376.8  (242.8-701.6) | 263.2  (158.2-471.7) | 444.8  (314.6-717.5) | 413.1  (309.2-704.6) |
|  | **Tunica muscularis** | **705.7**  **(411.3-1197.6)** | **754.7**  **(493.8-1403.8)** | **568.6**  **(327.8-930.7)** | **917.1**  **(639.7-1352.7)** | **785.6**  **(560.8-1429.8)** |
|  | **Whole esophageal wall** | **1668.5**  **(1181.3-2395.1)** | **1724.5**  **(1421.2-2744)** | **1965.9**  **(1194.9-2362.2)** | **1779.2**  **(1096.9-2976.6)** | **1687.8**  **(1160.6-2450.8)** |

All values are given as median (min-max)

European roe deer 1


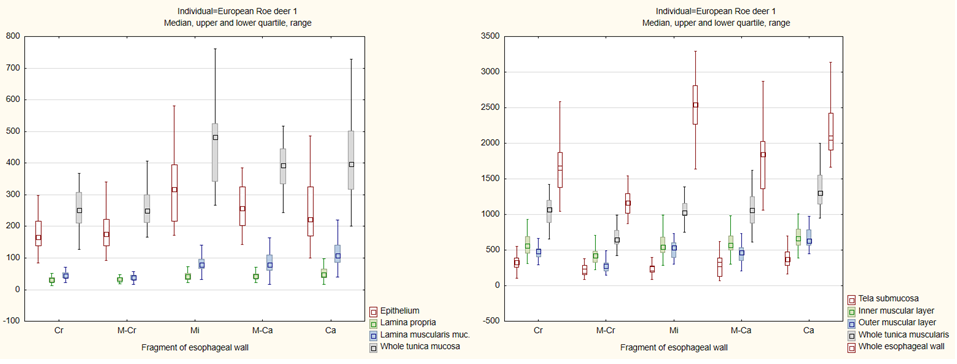


European roe deer 2


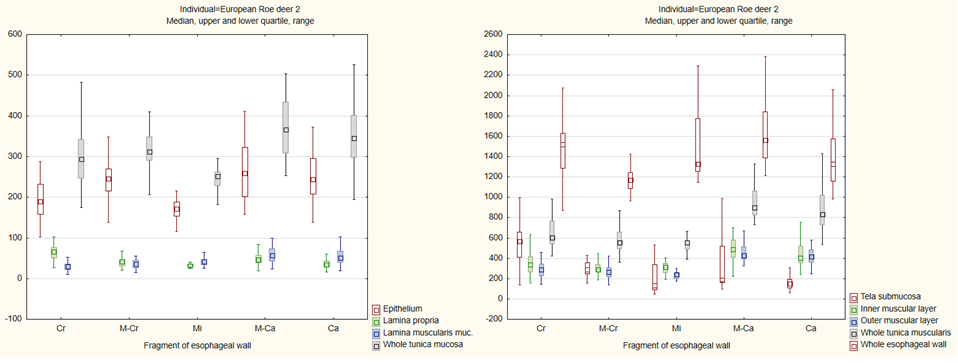


European roe deer 3


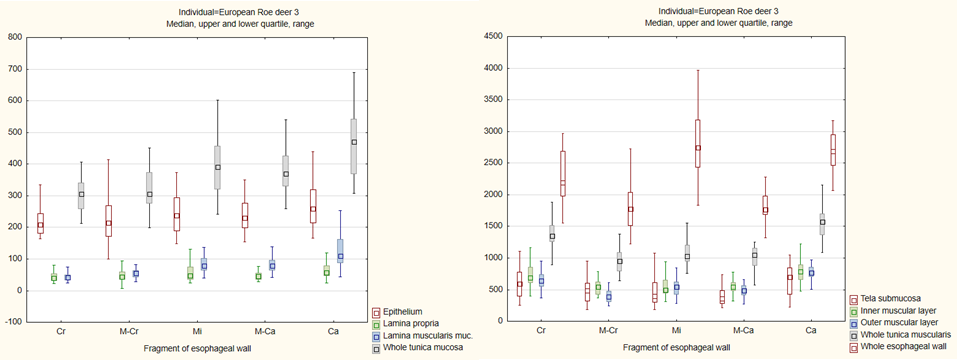


European roe deer 4


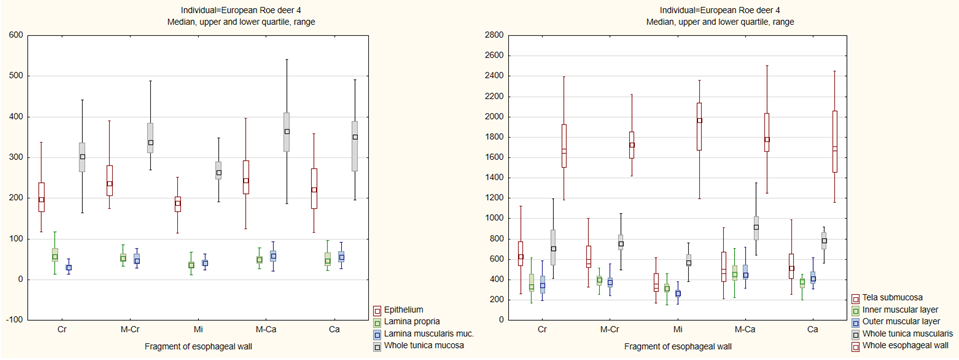

Supplement: Supplementary file 1 — SUPPORTING INFORMATION [file VMS3-7-1743-s001.doc]
